# Coronavirus Nsp5‑mediated dual‑site cleavage of GSDMA modifies its antiviral and proinflammatory functions

**DOI:** 10.1371/journal.ppat.1014602

**Published:** 2026-09-16

**Authors:** Chenyu Li, Yuxi Zhao, Yuchen Zhang, Shuai Li, Longfei Chen, Xiangfei Xu, Tong Ding, Yuanqing Wang, Xiang Gao, Zhaoyu Lin, Guanning Su, Ankang Hu, Liurong Fang, Qi Su, Quangang Chen, Yanrong Zhou, Shaobo Xiao

**Affiliations:** 1 National Key Laboratory of Agricultural Microbiology, College of Veterinary Medicine, Huazhong Agricultural University, Wuhan, China; 2 The Key Laboratory of Preventive Veterinary Medicine in Hubei Province, Cooperative Innovation Center for Sustainable Pig Production, Wuhan, China; 3 State Key Laboratory of Pharmaceutical Biotechnology, Ministry of Education Key Laboratory of Model Animal for Disease Study, Jiangsu Key Laboratory of Molecular Medicine, Model Animal Research Center, National Resource Center for Mutant Mice of China, Nanjing Drum Tower Hospital, School of Medicine, Nanjing University, Nanjing, China; 4 Center of Animal Laboratory, Xuzhou Medical University, Xuzhou, China; 5 Department of Medicine and Therapeutics, The Chinese University of Hong Kong, Hong Kong SAR, China; Wuhan Institute of Virology Chinese Academy of Sciences, CHINA

## Abstract

Although Gasdermin A (GSDMA) drives inflammation by inducing pyroptosis, its specific role in antiviral defense remains unclear. Here we identify GSDMA as an immunomodulatory protein activated in response to coronavirus (CoV) infection. Specifically, CoV-encoded protease nsp5 cleaves GSDMA at two conserved glutamine sites, Q247 and Q187. Cleavage at Q247 liberates an active N-terminal fragment (GSDMA_1–247) that triggers pyroptosis, promotes inflammation, and restricts viral replication. In contrast, cleavage at the alternative site Q187 attenuates this function. Using *Gsdma*^-/-^ mice, we show that GSDMA deficiency increases viral loads but reduces inflammation, tissue damage, and mortality upon infection. These findings suggest that disease severity is driven more by inflammation than by viral load. Our findings reveal a novel mechanism of antiviral immunity and inflammatory regulation via CoV nsp5-mediated dual cleavage of GSDMA, highlighting a potential target for combined antiviral and anti-inflammatory therapies.

## Author summary

Coronaviruses continue to threaten global health, as demonstrated by recent outbreaks. Gasdermin A (GSDMA) is a host protein involved in pyroptosis, which is an inflammatory cell death. In this study, we demonstrate that the coronavirus 3C-like protease cleaves GSDMA at two distinct sites, generating fragments with different functional properties. One cleavage product enhances inflammation and limits viral replication, whereas the other does not exhibit these activities. Our findings suggest that coronaviruses 3C-like proteases may regulate cleavage at these two sites to influence the relative abundance of the resulting fragments, thereby balancing antiviral defense and inflammatory responses. Using a mouse model, we further demonstrate that loss of GSDMA results in higher viral loads but reduced inflammatory pathology and improved survival. These observations indicate that disease severity may be more closely linked to the magnitude of the inflammatory response than to viral burden alone. Together, our work highlights the importance of targeting both viral replication and dysregulated inflammation in therapeutic strategies.

## Introduction

Coronaviruses (CoVs) represent a significant and ongoing threat to public health, as exemplified by recent pandemics. This expansive viral family, characterized by its broad host range and tissue tropism, can cause diverse diseases across multiple organ systems [1–3]. A key determinant of CoV pathogenicity is the virus’s ability to manipulate host cell machinery, often through the action of virally encoded proteins [4–11]. Among these, the highly conserved nonstructural protein 5 (nsp5), or main protease, is essential for processing viral polyproteins and has been increasingly shown to cleave host proteins to subvert innate immune responses [4–7,12–14].

One of the critical host defense mechanisms is pyroptosis, an inflammatory form of programmed cell death executed by gasdermin (GSDM) family proteins [7,15–17]. Host proteolytic cleavage activates most GSDMs, releasing a pore-forming N-terminal fragment [15–19]. Although avian GSDMA has been shown to be cleaved by caspase-1, no mammalian host protease has been reported to cleave GSDMA [20]. While certain bacterial proteases are known to activate GSDMA [21–23], direct cleavage and activation by viral proteases remain largely unexplored. Intriguingly, a prior study from our laboratory identified GSDMA as a putative substrate for nsp5 from porcine epidemic diarrhea virus (PEDV), an α-CoV [24]. This finding, coupled with the known role of nsp5 in cleaving host immune signaling molecules, led us to the following stepwise hypotheses: First, given the conserved function of nsp5 across CoVs, we hypothesized that GSDMA is a conserved substrate of the CoV nsp5. Second, since the predicted cleavage occurs at Q247, and previous studies have shown that GSDMA induces pyroptosis when cleaved at Q247, we reasoned that nsp5-mediated cleavage of GSDMA would trigger pyroptosis. Third, based on the dual nature of inflammatory responses—which can be both antiviral and pathological—we postulated that this GSDMA-dependent pyroptosis would exert a complex, dual role during infection: curbing viral replication but also potentially contributing to inflammatory tissue damage.

To test these hypotheses, we investigated the interaction between GSDMA and nsp5 across representative mammalian CoVs. We first confirmed the cleavage events, mapped the specific sites, and characterized the functional consequences of the resulting fragments *in vitro*. Then, *Gsdma*^-/-^ mice were utilized to define the net impact of GSDMA on host outcomes during *in vivo* CoV infection, dissecting its effects on viral control, inflammation, and overall disease pathogenesis. This work unveils a novel virus-host interface where a conserved viral protease directly activates a host pyroptotic effector, revealing a critical and nuanced role for GSDMA in determining the balance between antiviral defense and inflammatory pathology.

## Results

### Coronavirus Nsp5 cleaves GSDMA between residues Q187 and Q247

**Fig 1:**
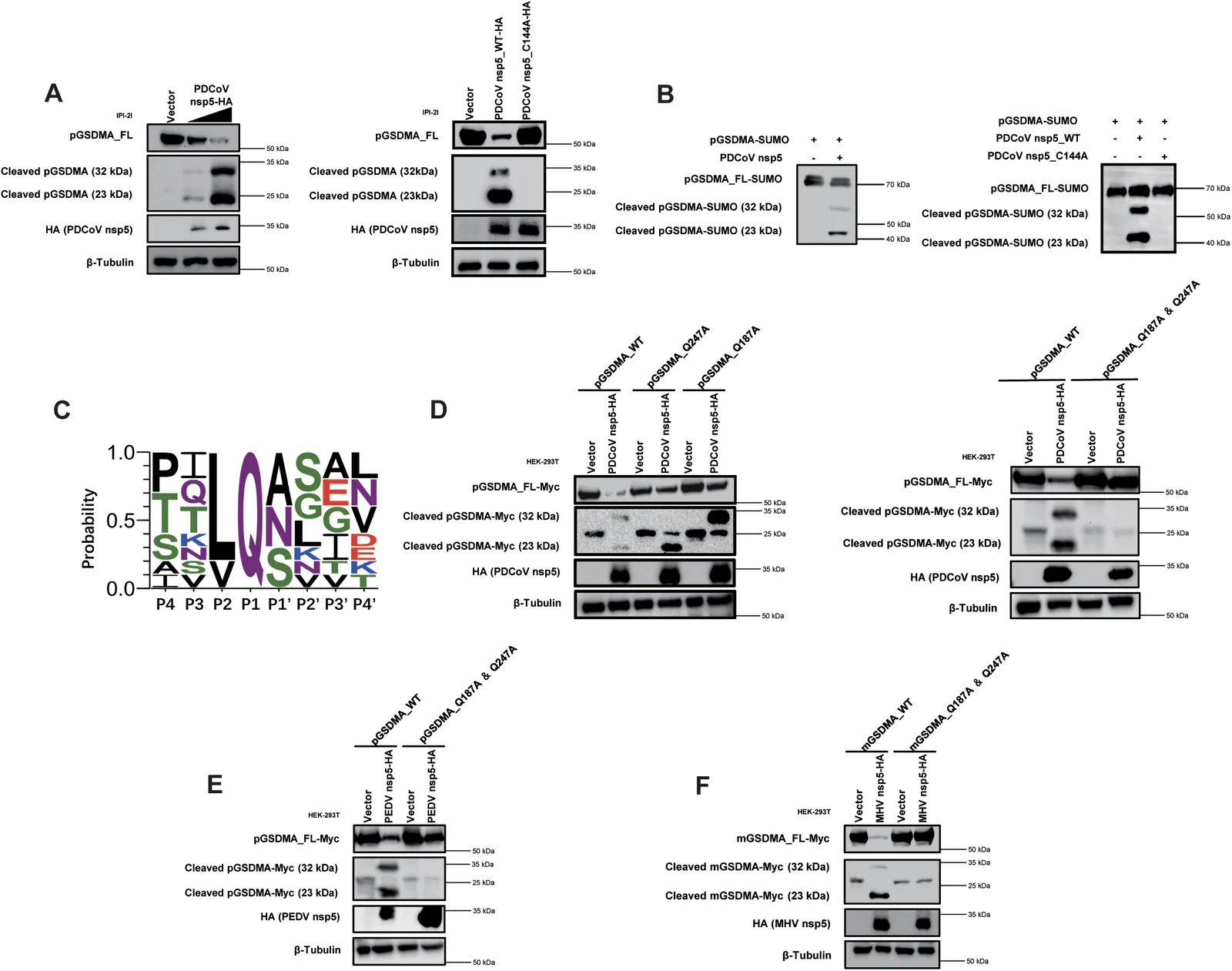
CoV nsp5 cleaves GSDMA at residues Q187 and Q247. **(A)** IPI-2I cells were transfected with eukaryotic expression plasmids encoding HA-tagged PDCoV nsp5_WT or PDCoV nsp5_C144A. Cells were lysed at 48 h post-transfection (hpt), and the expression levels of endogenous full-length porcine GSDMA (pGSDMA_FL) and cleaved pGSDMA were determined by immunoblotting using an anti-pGSDMA antibody. β-Tubulin was used as a loading control. **(B)** Recombinant SUMO-tagged pGSDMA protein (pGSDMA_FL-SUMO; 12.5 μg) was incubated with purified PDCoV nsp5_WT or PDCoV nsp5_C144A proteins (1 μg each) for 30 min. The levels of pGSDMA_FL-SUMO and cleaved pGSDMA-SUMO were analyzed by immunoblotting using an anti-pGSDMA antibody. **(C)** Amino acid sequence logos of the PDCoV nsp5 auto-cleavage sequences (nsp5-nsp16) were created using PSSMSearch, and the height of the letters represents the relative frequency of the amino acids. **(D)** Cells were co-transfected with eukaryotic expression plasmids encoding HA-tagged PDCoV nsp5 and Myc-tagged pGSDMA_FL (wild-type pGSDMA_FL, abbreviated as pGSDMA_WT) or pGSDMA_FL mutants (pGSDMA_Q187A, pGSDMA_Q247A, and pGSDMA_Q187A&Q247A). **(E)** Cells were co-transfected with eukaryotic expression plasmids encoding HA-tagged PEDV nsp5 and Myc-tagged pGSDMA_WT or pGSDMA_Q187A&Q247A. **(F)** Cells were co-transfected with eukaryotic expression plasmids encoding HA-tagged MHV nsp5 and Myc-tagged murine GSDMA (mGSDMA)_WT or mGSDMA_Q187A&Q247A. **(D-F)** HEK-293T cells were lysed at 48 hpt, and the expression of GSDMA_FL and cleaved GSDMA was determined by immunoblotting using an anti-Myc antibody. β-Tubulin was used as a loading control.

Our prior substrate degradome screen identified members of the GSDM family, specifically GSDMA and GSDMD, as putative targets of CoV nsp5 [24]. Subsequent studies by our group and others confirmed nsp5‑mediated cleavage of GSDMD, but whether nsp5 also cleaves GSDMA remains uninvestigated [7,25–27]. To clarify this, we first transfected plasmids encoding the nsp5 of porcine deltacoronavirus (PDCoV), a coronavirus capable of infecting multiple species, into IPI-2I cells (porcine ileum epithelial cells), a cell line derived from porcine ileum epithelial cells, which are the primary target cells of PDCoV. The results showed that overexpression of wild-type PDCoV nsp5 (PDCoV nsp5_WT), but not its catalytically inactive mutant (PDCoV nsp5_C144A), led to a reduction of full-length porcine GSDMA (pGSDMA_FL) protein levels and the emergence of two smaller cleavage fragments: one of ~32 kDa and the other of ~23 kDa (Fig 1A), suggesting that PDCoV nsp5 cleaves pGSDMA in an enzymatic activity-dependent manner. To further determine whether PDCoV nsp5 directly cleaves pGSDMA, we expressed and purified recombinant pGSDMA-SUMO, PDCoV nsp5_WT, and PDCoV nsp5_C144A proteins. Upon mixing the purified proteins, we observed the emergence of cleavage fragments similar in size to those detected in the cellular experiments in the nsp5_WT group, but not in the nsp5_C144A group (Fig 1B), confirming that PDCoV nsp5 can directly cleave pGSDMA in a manner dependent on its enzymatic activity.

Next, we aimed to identify the cleavage sites of pGSDMA targeted by PDCoV nsp5. Analysis of the sequences surrounding the cleavage sites in the viral polyprotein, a natural substrate of PDCoV nsp5, revealed that PDCoV nsp5 exhibits a strong preference for substrates containing a glutamine (Q) residue at the P1 position immediately adjacent to the cleavage site (Fig 1C). Given that the cleavage of pGSDMA by PDCoV nsp5 produces fragments ~23 kDa and ~32 kDa in size (Fig 1A), we hypothesized that PDCoV nsp5 may cleave pGSDMA at residues Q187 and Q247. Supporting this hypothesis, we observed that the mutation of Q187 to alanine (Q187A) blocked the emergence of the ~ 23 kDa fragment, while the mutation of Q247 to alanine (Q247A) prevented the formation of the ~ 32 kDa fragment (Fig 1D). These results demonstrate that PDCoV nsp5 cleaves pGSDMA specifically at residues Q187 and Q247.

Notably, temporal analysis revealed that the ~ 32 kDa fragment (GSDMA_1–247) emerged as the predominant cleavage product at early time points (S1A Fig), whereas extended incubation resulted in a progressive increase in the ~ 23 kDa fragment (GSDMA_1–187) (Fig 1B). This observation prompted us to hypothesize that nsp5 may not only cleave GSDMA_FL at Q187 but also facilitate a secondary cleavage of GSDMA_1–247 at Q187. Consistently, co-overexpression of GSDMA_1–247 and nsp5 in HEK-293T cells (human embryonic kidney 293 cells) led to the generation of a ~ 23 kDa fragment corresponding to GSDMA_1–187 (S1B Fig).

Homology analysis revealed that residues Q187 and Q247 in GSDMA sequences are highly conserved across various species (S1C Fig). Considering that the P1 site of CoV nsp5 shows a strong preference for Q, we speculated that CoV nsp5 may possess the potential to cleave GSDMA across different species. To test this speculation, we first investigated whether PDCoV nsp5 could cleave the GSDMA of other PDCoV-susceptible species besides porcine [28–32]. Using Huh-7 cells (human hepatocellular carcinoma cells) and iBMDMs (immortalized murine bone marrow-derived macrophages), we found that PDCoV nsp5 cleaved both human GSDMA (hGSDMA) and murine GSDMA (mGSDMA), generating fragments of ~32 kDa and ~23 kDa (S1D and S1E Fig). We then examined whether this cleavage of GSDMA is a shared characteristic of nsp5 from other mammalian CoVs. To this end, we analyzed nsp5 from α-CoVs (PEDV and human coronavirus NL63 [HCoV-NL63]) and β-CoVs (severe acute respiratory syndrome coronavirus 2 [SARS-CoV-2], Middle East respiratory syndrome coronavirus [MERS-CoV], HCoV-HKU1, HCoV-OC43, and murine hepatitis virus [MHV]) using cell lines derived from species susceptible to each corresponding CoV. We observed that, in every case, nsp5 from the tested CoVs cleaved GSDMA of their respective susceptible species, producing two cleavage fragments similar in size to those observed with PDCoV nsp5 (S1F–S1L Fig). To further confirm these findings, we selected PEDV (α-CoV) and MHV (β-CoV) as representative CoVs and demonstrated that, like PDCoV nsp5, both PEDV and MHV nsp5 cleaved GSDMA at residues Q187 and Q247 (Fig 1E and 1F). Collectively, these results indicate that cleavage of GSDMA at residues Q187 and Q247 is a conserved feature of nsp5 across mammalian CoVs.

### Infections of CoVs Induce the Cleavage of GSDMA Both *In Vivo* and *In Vitro*

**Fig 2:**
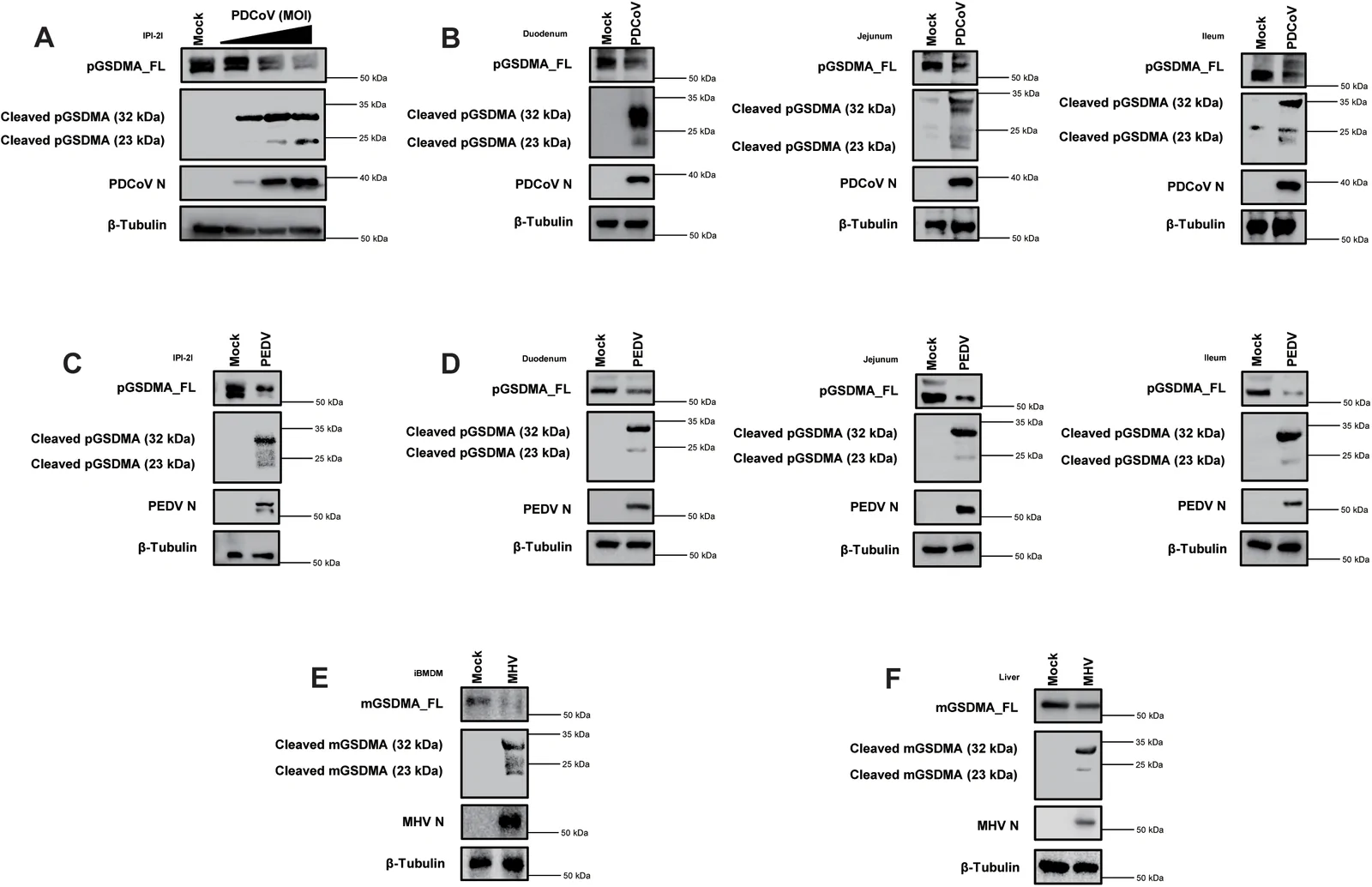
CoV infections cause the cleavage of GSDMA both *in vivo* and *in vitro.* **(A and C)** IPI-2I cells were infected with PDCoV (0.05, 0.1, and 0.5 MOI) **(A)** or with PEDV (0.25 MOI) **(C)**, and lysed for immunoblotting at 24 hours post-infection (hpi). (B and D) Small intestinal tissues from piglets, either mock-infected or infected with PDCoV **(B)** or PEDV **(D)**, were harvested and lysed for immunoblotting. **(E)** iBMDMs were infected with MHV (0.5 MOI) and lysed for immunoblotting at 8 hpi. **(F)** Liver tissues from mice, either mock-infected or infected with MHV, were harvested and lysed for immunoblotting. **(A-F)** Endogenous GSDMA_FL and cleaved GSDMA were assessed using anti-GSDMA antibodies. β-Tubulin was used as a loading control.

To determine whether GSDMA is cleaved during CoV infections, we initially assessed the effects of PDCoV infection on GSDMA using IPI-2I cells and porcine small intestinal tissues, including the duodenum, jejunum, and ileum. Our results revealed that PDCoV infection led to a reduction in the levels of pGSDMA_FL protein, accompanied by the appearance of two cleavage fragments (~32 kDa and ~23 kDa) (Fig 2A and 2B). These observations, noted both *in vivo* and *in vitro*, align with the results from the overexpression of PDCoV nsp5 in IPI-2I cells. Similarly, infections with PEDV or MHV in IPI-2I cells or iBMDMs, as well as in porcine small intestinal tissues or mouse liver, also resulted in the generation of two cleavage fragments of both pGSDMA and mGSDMA (Fig 2C to 2F). These findings collectively indicate that CoV infections lead to the cleavage of GSDMA both *in vivo* and *in vitro*.

### CoV Nsp5 Cleaves GSDMA at Q247 to Induce Pyroptosis and Inflammatory Responses

**Fig 3:**
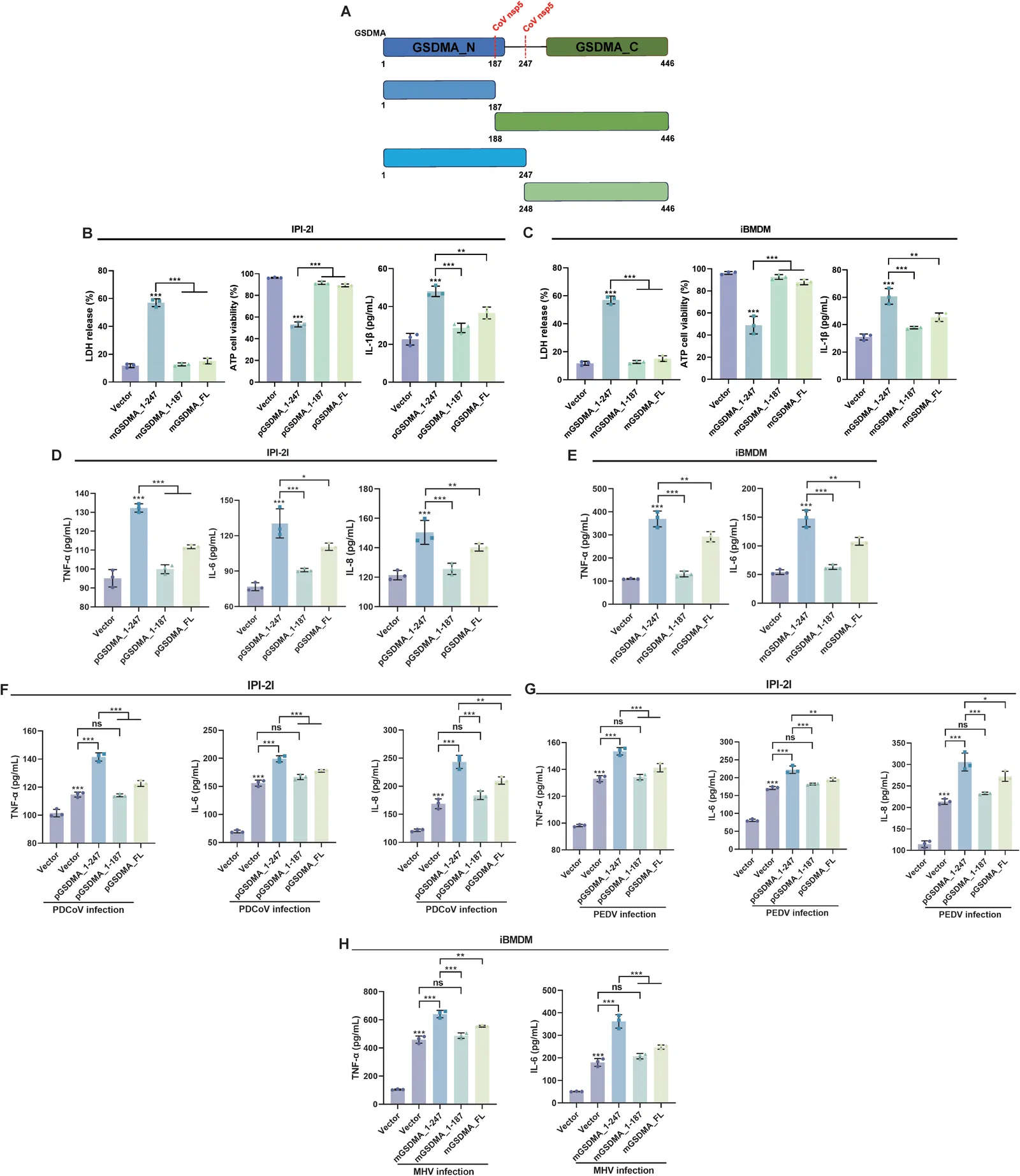
GSDMA_1-247 contributes to GSDMA-mediated inflammatory responses. **(A)** Schematic representation of GSDMA and its cleavage fragments induced by CoV nsp5. **(B, D, F, and G)** IPI-2I cells were transfected with eukaryotic plasmids encoding pGSDMA_1-247, pGSDMA_1-187, and pGSDMA_FL. **(B and D)** Cell supernatants and/or cell lysates were then collected at 48 hpt. **(F and G)** Cells were then infected with PDCoV (0.5 MOI) **(F)** or PEDV (0.25 MOI) **(G)** for an additional 24 h, after which the cell supernatants were collected. **(B)** LDH release and IL-1β levels in the cell supernatants, as well as intracellular ATP cell viability, were analyzed using assay kits. **(D, F, and G)** The protein levels of porcine proinflammatory cytokines (TNF-α, IL-6, and IL-8) in the cell supernatants were assessed through ELISA. **(C, E, and H)** iBMDMs were transfected with eukaryotic plasmids encoding mGSDMA_1-247, mGSDMA_1-187, or mGSDMA_FL. **(C, E)** Cell supernatants and/or cell lysates were collected at 48 hpt. (H) Cells were subsequently infected with MHV (0.5 MOI) for an additional 8 h, and the cell supernatants were then collected. **(C)** LDH release and IL-1β levels in the cell supernatants, as well as intracellular ATP cell viability, were analyzed using assay kits. **(E and H)** The protein levels of murine proinflammatory cytokines (TNF-α and IL-6) were assessed. **(B-H)** Data analysis was performed using one-way ANOVA. Values are shown as the mean ± SD from three independent experiments. *, *p* < 0.05; **, *p* < 0.01; ***, *p* < 0.001; ns, not significant.

GSDMA, a member of the GSDM family, is known to mediate pyroptosis. Consequently, we sought to investigate the contributions of GSDMA cleavage fragments—specifically, GSDMA_1–247 (~32 kDa) and GSDMA_1–187 (~23 kDa) (Fig 3A)—to the induction of GSDMA-mediated pyroptosis. To clarify this, we overexpressed these two cleavage fragments from porcine and murine sources in IPI-2I cells and iBMDMs. As shown in Fig 3B and 3C, the results revealed that GSDMA_1–247 significantly promoted lactate dehydrogenase (LDH) release (a marker of lytic cell death), decreased cell viability as measured by adenosine triphosphate production, and elevated interleukin-1β (IL-1β) protein levels (a biomarker of pyroptosis). In contrast, GSDMA_1–187 showed no significant effects on LDH release, IL-1β protein levels, or cell survival. Additionally, using GSDMA-specific siRNA (siGSDMA) to knock down GSDMA expression (S2A and S2B Fig), we observed that GSDMA plays an essential role in triggering pyroptosis during CoV infections (S2C to S2E Fig). Based on a comprehensive analysis of these results, we conclude that CoVs exploit GSDMA to trigger pyroptosis by cleaving GSDMA via viral nsp5, resulting in the production of cleavage fragments GSDMA_1–187 and GSDMA_1–247, with GSDMA_1–247 acting as a key inducer of pyroptosis, while GSDMA_1–187 lacks this capability.

Given that pyroptosis is a form of inflammatory cell death, we further investigated whether GSDMA and its cleavage fragments contribute to the inflammatory responses triggered by CoV infections. Using ELISA, we found that knockdown of GSDMA significantly reduced the protein levels of proinflammatory cytokines induced by CoV infections, including PDCoV, PEDV, and MHV (S3A to S3C Fig). These results suggest that GSDMA is critical for the induction of proinflammatory cytokines during CoV infections. We then assessed whether GSDMA cleavage fragments specifically contribute to this proinflammatory effect. Our results showed that the overexpression of GSDMA_1–247 significantly upregulated the levels of proinflammatory cytokines, while the overexpression of GSDMA_1–187 exhibited no significant effect on proinflammatory cytokine levels (Fig 3D and 3E). A similar regulatory pattern was observed under conditions of CoV infections (Fig 3F to 3H). These findings highlight that GSDMA_1–247, rather than GSDMA_1–187, plays a dominant role in triggering inflammatory responses.

### GSDMA_1–247 Fragment Antagonizes the Replication of CoVs

**Fig 4:**
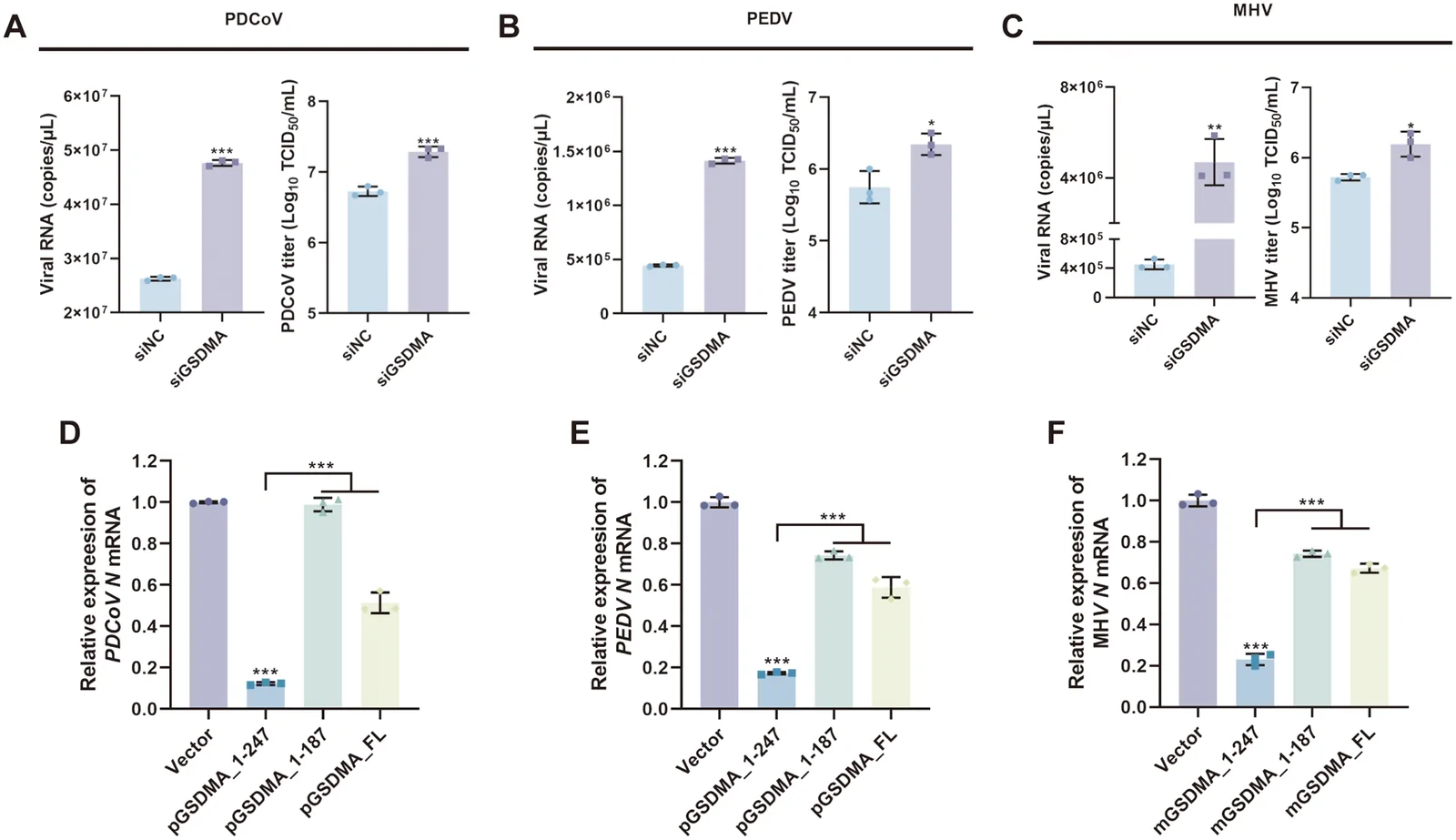
GSDMA_1-247 is responsible for the antiviral activity of GSDMA. **(A-C)** IPI-2I cells **(A and B)** or iBMDMs **(C)** were transfected with GSDMA-specific siRNA (siGSDMA) or control siRNA (siNC) for 36 h, followed by infection with PDCoV (0.5 MOI) for an additional 24 h **(A)**, PEDV (0.25 MOI) for an additional 24 h **(B)**, or MHV (0.5 MOI) for an additional 8 h **(C)**. (D-F) IPI-2I cells **(D and E)** or iBMDMs **(F)** were transfected with eukaryotic expression plasmids encoding GSDMA_1-247, GSDMA_1-187, or GSDMA_FL. At 48 hpt, cells were subsequently infected with PDCoV (0.5 MOI) for an additional 24 h **(D)**, PEDV (0.25 MOI) for an additional 24 h **(E)**, or MHV (0.5 MOI) for an additional 8 h **(F)**. **(A-F)** Total RNA extracted from cell lysates was analyzed by RT‑qPCR, and viral titers in cell supernatants were determined by the TCID₅₀ assay. Data analysis was performed using Student’s *t*-test or one-way ANOVA. Viral RNA copy number was measured using RT-qPCR. Values are shown as the mean ± SD from three independent experiments. *, *p* < 0.05; **, *p* < 0.01; ***, *p* < 0.001.

We next investigated whether the pyroptotic functions of GSDMA and its cleavage fragments during CoV infections are linked to their potential regulation of viral replication. First, we examined the impact of GSDMA on CoV replication. Using siGSDMA, we found that knockdown of pGSDMA in IPI-2I cells or mGSDMA in iBMDMs significantly increased the viral load of various CoVs, including PDCoV, PEDV, and MHV (Fig 4A to 4C). Conversely, overexpression of both pGSDMA_FL and mGSDMA_FL significantly inhibited viral proliferation (Fig 4D to 4F), indicating that GSDMA exhibits antiviral activity against CoVs. Building on these findings, we evaluated whether nsp5-mediated cleavage contributes to the antiviral activity of GSDMA. As illustrated in Fig 4D to 4E, the GSDMA_1–247 fragment demonstrated significant antiviral capacity against CoVs. In contrast, the GSDMA_1–187 fragment exhibited significantly weaker anti-CoV activity compared to GSDMA_1–247 (Fig 4D to 4E). These results suggest that GSDMA functions as an antagonist of CoV proliferation, with its antiviral effects predominantly attributable to the GSDMA_1–247 fragment, rather than the GSDMA_1–187 fragment, generated by CoV nsp5-mediated cleavage. Collectively, our study reveals that while GSDMA promotes CoV-induced inflammatory responses and pyroptotic cell death, it simultaneously inhibits viral proliferation, suggesting that the pyroptotic and proinflammatory activities of GSDMA are mechanistically independent of its antiviral effects. To further elucidate the basis for the differential antiviral effects of GSDMA_FL and its cleavage fragments (GSDMA_1–187 and GSDMA_1–247), we examined their impact on interferon signaling. Our results revealed that while GSDMA upregulated the transcription of interferon-β (*IFNB1*/*Ifnb1*) and interferon-stimulated genes (*ISG*s/*Isg*s), the GSDMA_1–247 fragment, rather than GSDMA_1–187, induced significantly higher expression levels of these genes (S4A and S4B Fig). These findings suggest that the antiviral effects of GSDMA_FL and GSDMA_1–247 may be mediated by their enhancement of IFN signaling. Nevertheless, the antiviral activity of GSDMA is likely multifactorial. We cannot exclude the possibility that additional mechanisms contribute to its antiviral effects. For example, GSDMA may inhibit viral replication through the induction of pyroptotic cell death.

### *Gsdma* knockout Prevents Mice from lethal CoV infections

**Fig 5:**
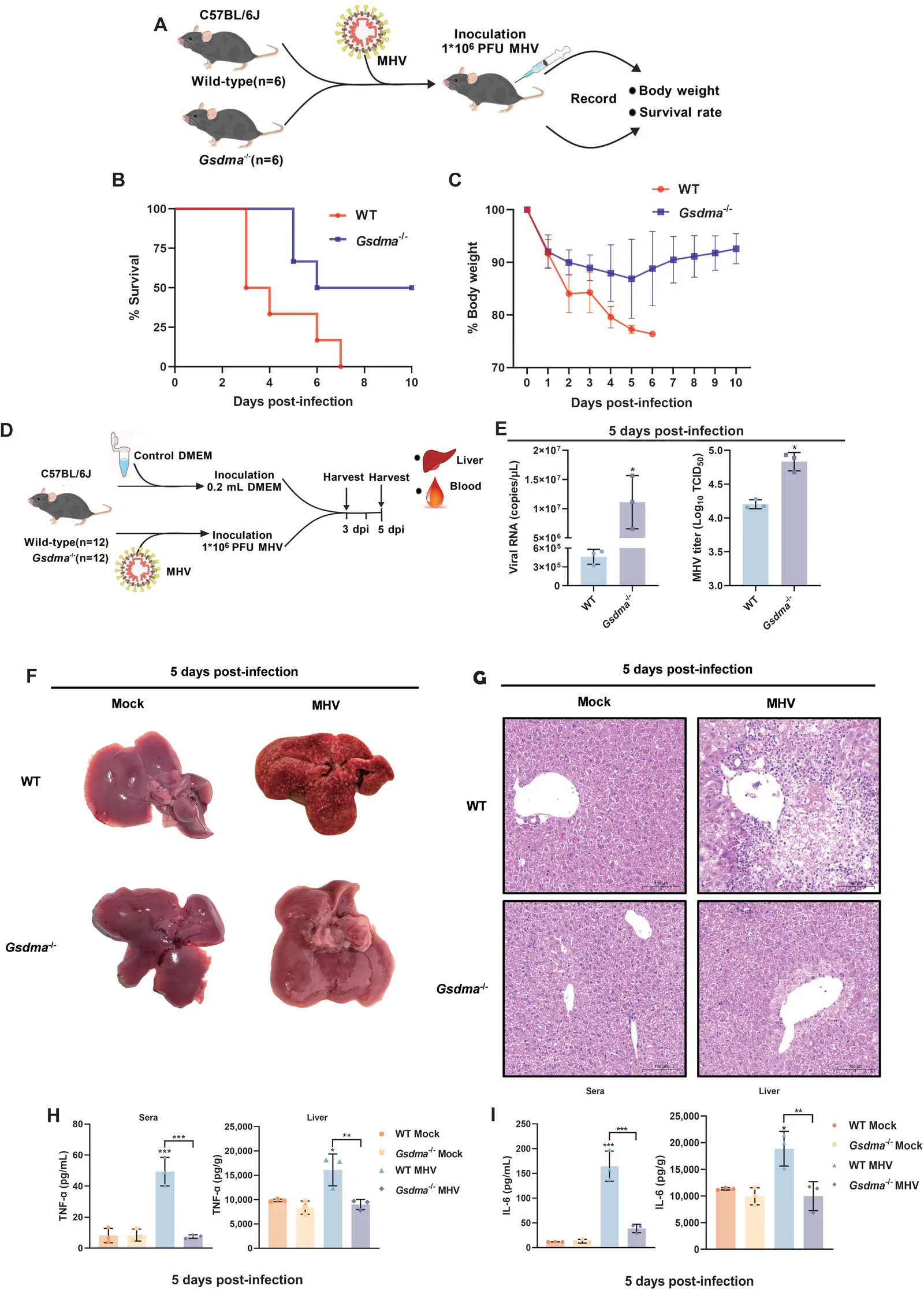
GSDMA knockout reduces inflammation and mortality *in vivo.* **(A)** Schematic representation of the animal experiment protocol. Groups of six WT or *Gsdma*^-/-^ mice were inoculated with 1 × 10^6^ PFU of MHV via intraperitoneal injection. The mortality **(B)** and body weight changes **(C)** of the mice were recorded over a consecutive ten-day period. **(D)** Schematic representation of the animal experiment protocol. Groups of six WT or *Gsdma*^-/-^ mice were inoculated with either DMEM as a negative control or 1 × 10^6^ PFU of MHV via intraperitoneal injection. Three mice from each group were euthanized at 3 and 5 dpi, respectively. Liver tissues were collected for virus titration, pathological analysis, and ELISA, while blood samples were collected for ELISA. (E-G) Liver tissues from mice euthanized at 5 dpi were analyzed for viral RNA copy number and titers in L929 cells **(E)**, gross lesions **(F)**, and pathological changes **(G)**. (H and I) Serum and liver tissues from mice euthanized at 5 dpi were collected to assess TNF-α **(H)** and IL-6 **(I)** protein levels via ELISA. **(E, H, and I)** Data analysis was performed using Student’s *t*-test **(E)** or one-way ANOVA **(H and I)**. Values are shown as the mean ± SD. *, *p* < 0.05; **, *p* < 0.01; ***, *p* < 0.001.

Given that GSDMA induces pyroptosis, promotes inflammatory responses, and inhibits viral proliferation during CoV infections, we speculated that GSDMA knockout might affect the pathogenicity of CoVs. The mouse genome encodes three GSDMA paralogs (*Gsdma1*, *Gsdma2*, and *Gsdma3*) with potential functional redundancy. To address this, we employed *Gsdma1*/*2*/*3* triple-knockout (*Gsdma*^-/-^) mice to phenocopy the single *Gsmda* gene typically found in other mammals. Both *Gsdma*^-/-^ mice and WT mice were intraperitoneally injected with MHV at a dose of 1 × 10^6^ PFU per mouse (Fig 5A). We observed that WT mice (n = 6) began to succumb to the infection on day 3 post-infection, with all six mice dead by day 7. In contrast, *Gsdma*^-/-^ mice (n = 6) started dying on day 5 post-infection, with three of the six mice surviving for the entire experimental duration of 10 days (Fig 5B). Additionally, the body weights of WT mice continuously decreased after MHV infections until mortality occurred. Although *Gsdma*^-/-^ mice experienced gradual weight loss during the first five days post-infection (dpi), the surviving *Gsdma*^-/-^ mice began to regain weight from 6 dpi. Notably, the body weights of *Gsdma*^-/-^ mice remained consistently higher than those of WT mice throughout the experimental period (Fig 5C).

To further evaluate the effects of GSDMA on the proliferation and pathogenicity of CoVs, an additional batch of WT and *Gsdma*^-/-^ mice were inoculated with MHV (Fig 5D). Western blot analysis revealed the presence of GSDMA cleavage fragments (~32 kDa and ~23 kDa) in MHV-infected WT mice but not in *Gsdma*^-/-^ mice (S5A Fig), consistent with the cleavage patterns observed in infected cell lines. Based on the mortality data, which revealed that WT and *Gsdma*^-/-^ mice began to succumb to the MHV infection on days 3 and 5 post-infection, respectively (Fig 5B), three mice from each group were euthanized at 3 dpi and 5 dpi (Fig 5D). Subsequently, viral RNA copies and titers in the livers were quantified using RT-qPCR and TCID_50_ assays. The results demonstrated that, consistent with the *in vitro* findings, the viral load in *Gsdma*^-/-^ mice was significantly higher than in WT mice at both 3 dpi and 5 dpi (Fig 5E and S5B Fig). Interestingly, despite the increased viral load observed in *Gsdma*^*-/-*^ mice, the pathological changes in these mice were less severe than those in WT mice. Specifically, visual inspection revealed that WT mice infected with MHV displayed greyish-white necrotic foci in the liver, which became progressively more numerous and pronounced as the infection advanced (Fig 5F and S5C Fig). In contrast, no visible necrotic foci were observed in the livers of *Gsdma*^-/-^ mice (Fig 5F and S5C Fig). Histopathological analysis provided further evidence of these differences. In WT mice, diffuse infiltration of lymphocytes and monocytes was observed within the portal areas and hepatic lobules at 3 dpi (S5D Fig); by 5 dpi, this inflammation had spread throughout the hepatic lobules, with normal liver structures largely replaced by inflammatory cells (Fig 5G). Conversely, *Gsdma*^-/-^ mice exhibited only localized inflammatory infiltration in the portal areas and hepatic lobules at both 3 and 5 dpi, indicating a markedly attenuated inflammatory response. Consistent with these pathological observations, GSDMA knockout significantly inhibited pyroptosis and inflammatory responses *in vivo* during MHV infection. This was evidenced by reduced protein levels of IL-1β, a canonical biomarker of pyroptosis, and proinflammatory cytokines (TNF-α and IL-6) in the livers and sera of MHV-infected *Gsdma*^-/-^ mice compared to those of MHV-infected WT mice (Fig 5H and 5I and S5E to S5G Fig).

## Discussion

Mammalian GSDM family members comprise GSDMA, GSDMB, GSDMC, GSDMD, GSDME, and pejvakin (PJVK). Among these, GSDMA, as an ancient member of the GSDM family, is considered the evolutionary ancestor of GSDMB, GSDMC, and GSDMD [20]. Intriguingly, avian GSDMs consist solely of GSDMA, GSDME, and PJVK [20], suggesting that avian GSDMA may have retained the biological functions associated with GSDMB, GSDMC, and GSDMD in mammals. Supporting this hypothesis, in mammals, activated NLRP3 facilitates the release of mature IL-1β by cleaving GSDMD through caspase-1 [15]. In contrast, in birds, activated NLRP3 achieves the similar function by cleaving GSDMA via caspase-1 [20,33]. This indicates a functional parallel between avian GSDMA and mammalian GSDMD, suggesting that GSDMA in birds may substitute for GSDMD by adopting a comparable role in inflammasome activation and cytokine release. Collectively, these findings imply that mammalian GSDMA likely fulfills diverse biological functions.

Research on mammalian GSDMA remains limited compared to that on GSDMD and GSDME. In the present study, we demonstrate that CoV infection induces GSDMA-mediated pyroptosis in a nsp5-dependent manner. Notably, knockout of GSDMA does not completely abrogate pyroptosis (S2 and S5E Fig), suggesting that additional gasdermin family members may also contribute to CoV‑induced pyroptotic cell death. Consistent with this notion, our previous work has shown that PDCoV can trigger GSDME-mediated pyroptosis [7]. In addition, GSDMB has also been reported to participate in CoV-induced pyroptosis [34]. However, the precise contributions and regulatory mechanisms of different gasdermins during CoV infection remain to be defined.

Identifying the proteases responsible for cleaving GSDMs is crucial for understanding their functions and the regulatory mechanisms of pyroptosis. Previous studies have primarily focused on the cleavage of GSDMs by host proteases, such as caspases and granzymes [15–19,35]. However, recent findings have revealed the ability of pathogen-derived proteases to cleave GSDMs as well [8,21,22,36,37]. Notably, for GSDMA, *Streptococcus* protease SpeB has been reported to cleave it, thereby inducing pyroptosis [21,22]. However, no viral proteases have been documented to cleave GSDMA to directly induce pyroptosis. In this study, we demonstrate that viral proteases are capable of cleaving GSDMA to induce pyroptosis and this phenomenon is commonly observed in mammalian CoVs. Interestingly, during our investigations, recent work by Yin et al. reported that the 3C-like proteases (3CL^pro^) of Seneca Valley virus (SVV), a small RNA virus within the *Picornaviridae* family, can also cleave porcine and human GSDMA [38]. Their results revealed that the SVV 3CL^pro^ cleaves GSDMA exclusively at residue Q187, producing the fragment GSDMA_1–187 that inhibits pyroptosis. This contrasts with our findings, where the nsp5 of mammalian CoVs cleaves GSDMA at both residues Q247 and Q187. This is the first report demonstrating that GSDMA can be cleaved at two distinct sites, as previous studies have only identified single-site cleavage of GSDMA. More importantly, the resulting fragment GSDMA_1–247, unlike GSDMA_1–187, promotes pyroptosis. These results underscore the complexity of GSDMA regulation by viral proteases, suggesting that different viral proteases may employ diverse regulatory mechanisms and exert distinct effects on GSDMA.

Previously, we and other researchers have demonstrated that CoV nsp5 cleaves viral polyproteins as well as certain host proteins, such as NEMO, TRAF2/3, and STAT2, to promote viral replication [4,5,24,39–41]. However, in this study, we discovered that mammalian CoV nsp5 cleaves GSDMA, a protein with both *in vivo* and *in vitro* antiviral activity (Fig 4A to 4C, Fig 5E and S5A Fig), to generate the GSDMA_1–247 fragment, which stronger enhanced antiviral effects than GSDMA_FL. These findings suggest that nsp5-mediated cleavage of GSDMA represents a novel mechanism by which GSDMA potentiates its antiviral function. Given that GSDMA is highly expressed in barrier tissues such as the skin, which serve as the first line of defense against pathogen invasion [17,21,22], we hypothesize that GSDMA may act as a pathogen sensor, capable of perceiving specific pathogen-derived proteins, such as proteases, thereby leading to enhanced antiviral innate immune responses. Consequently, investigating the complex interplay between GSDMs and viruses is significant for better understanding host-virus interactions, providing valuable insights into the development of novel antiviral strategies with immunomodulatory potential.

Inflammation is also a crucial component of the innate immune response [42,43]. In this study, we found that, in addition to promoting IFN signaling, the GSDMA_1–247 fragment can also activate the inflammatory response. However, it is well known that the inflammatory response is a double-edged sword; low levels of inflammation may play a protective role in defending against pathogenic microbial infections, but excessive inflammation can be detrimental to the host. Our *in vivo* experimental results demonstrated that, following MHV infection, the inflammatory responses in WT mice were significantly stronger than those observed in *Gsdma*^*-/-*^ mice. Correspondingly, WT mice exhibited more severe clinical symptoms, pathological changes, and higher mortality rates compared to *Gsdma*^*-/-*^ mice. Interestingly, the viral loads in WT mice were lower than those in *Gsdma*^*-/-*^ mice, indicating that the severity of disease and mortality in infected animals is more closely associated with the level of inflammation rather than the viral load itself. This observation aligns with findings in bats, which carry high viral loads but inhibit Toll-like receptor 2-mediated immune signals, thereby preventing excessive inflammation and avoiding lesions or mortality following viral infections [44–46]. Our study suggests that, during viral infections, the primary factor contributing to mortality is the elevated levels of inflammation induced by the virus, rather than the viral load itself. These findings highlight the importance of combining antiviral drugs or antibodies with anti-inflammatory therapies to reduce viral load and mitigate tissue damage caused by excessive inflammation in the treatment of viral diseases.

Intriguingly, among the fragments of GSDMA generated by mammalian CoV nsp5-mediated cleavage, GSDMA_1–187 exhibited markedly diminished inflammation-inducing capacity compared to GSDMA_1–247. Given that nsp5 not only cleaves GSDMA_FL at Q187 but also mediates secondary cleavage of GSDMA_1–247 at Q187 (S1A Fig and S1B Fig), we speculated that this secondary cleavage may serve as a regulatory mechanism to prevent excessive inflammation. Specifically, nsp5 may initially preferentially cleave GSDMA at Q247 to produce GSDMA_1–247, thereby promoting inflammatory responses. Subsequently, as inflammation intensifies, nsp5 may further cleave GSDMA_1–247 at Q187 to generate GSDMA_1–187, a fragment with attenuated proinflammatory activity. This potential mechanism positions GSDMA as a critical regulatory node in balancing the host inflammatory response during CoV infection.

While this study establishes the critical dual-cleavage mechanism of GSDMA by nsp5, the precise temporal sequence and regulatory context governing cleavage at Q187 versus Q247 remain to be determined. Furthermore, our functional analysis relied on full-length GSDMA knockout models. The generation of cleavage site-specific mutant cell lines and animal models (e.g., GSDMA Q187A and Q247A) will be essential to unequivocally dissect the distinct biological functions of the full-length protein and its cleavage fragments during infection. Therefore, the potential stage-specific roles of GSDMA and its processed forms throughout the viral life cycle present a compelling avenue for future research. Moreover, we observed that CoV nsp5 triggers cell death and inflammatory responses (S6 Fig), comparable to those induced by GSDMA_1–247 overexpression and CoV infection; however, whether GSDMA primarily contributes to these effects requires further investigation.

In summary, our study provides the first evidence that viral proteases can trigger GSDMA-mediated pyroptosis, a conserved feature among mammalian CoVs. We identified that CoV nsp5 cleaves GSDMA at residues Q187 and Q247, revealing an unreported cleavage pattern of GSDMA. Functionally, the cleavage fragment GSDMA_1–247 drives pyroptosis, activates inflammatory responses, and inhibits viral replication, whereas GSDMA_1–187 lacks these functions. Utilizing *Gsdma*^*-/-*^ mice, we confirmed the antiviral, pyroptotic, and proinflammatory capacities of GSDMA *in vivo*, demonstrating that inflammation, rather than viral load, determines disease severity and mortality. Collectively, this study reveals a molecular mechanism by which GSDMA senses viral proteins to activate innate immunity and highlights inflammation as a key factor in CoV pathogenesis.

## Materials and methods

### Ethics statement

All procedures involving animal experiments were reviewed and approved in accordance with the Animal Experimental Ethical Inspection guidelines of the Laboratory Animal Center at Huazhong Agricultural University (Approval No: HZAUMO-2025–0263). Animal suffering and the number of animals used were minimized throughout the study.

### Cells and viruses

IPI-2I cells (porcine ileum epithelial cells), THP-1 cells (human monocytic cells), and Vero cells (African green monkey kidney cells) were obtained from the China Center for Type Culture Collection. LLC-PK1 cells (porcine kidney cells) and HEK-293T cells (human embryonic kidney 293 cells) were purchased from the American Type Culture Collection. iBMDMs (immortalized murine bone marrow-derived macrophages) were procured from SAE Biomed-tech (ZS) Company (China). L929 cells (mouse fibroblast cells) and Huh7 cells (human hepatocellular cells) were generously donated by Prof. Guiqing Peng and Wentao Li at Huazhong Agricultural University. IPI-2I, Huh-7, L929, and HEK-293T cells, as well as iBMDMs, were cultured in Dulbecco’s modified Eagle’s medium (DMEM; Gibco, USA). THP-1 cells were cultured in Roswell Park Memorial Institute 1640 medium (RPMI-1640; Sigma, USA). LLC-PK1 cells were cultured in Modified Eagle’s Medium (MEM; Hyclone, USA). All cells were cultured in media supplemented with 10% fetal bovine serum (FBS; TransGen, China) and incubated at 37°C with 5% CO_2_. The PDCoV strain CHN-HN-2014 (GenBank accession number: KT336560) and the PEDV strain AJ1102 (GenBank accession number: JX188454) were isolated from pigs suffering from severe diarrhea in China in 2014 and 2011, respectively [29,47]. MHV A59 strain (GenBank accession number: MF618253.1) was kindly provided by Prof. Guiqing Peng at Huazhong Agricultural University.

### Antibodies

Rabbit antibody against both murine GSDMA (mGSDMA) and human GSDMA (hGSDMA) was purchased from Abcam (UK). Mouse antibody against porcine GSDMA (pGSDMA) was developed in our laboratory, with its specificity validated through western blot analysis (S7A to S7C Fig). Rabbit antibody against β-Tubulin was obtained from ABclonal (China). Mouse anti-HA and anti-Myc antibodies were acquired from MBL (Japan). Mouse antibodies against the nucleocapsid (N) proteins of PDCoV and PEDV were prepared as previously described [29,48]. Mouse anti-MHV N antibody was generously provided by Prof. Zhaohui Qian from Peking Union Medical College.

### Plasmid and siRNA construction and transfection

Eukaryotic expression plasmids and prokaryotic expression plasmids containing the full-length cDNA of porcine GSDMA (pGSDMA_FL) were constructed by amplifying cDNAs from IPI-2I cells and subsequently cloning them into the pCAGGS vector with an N-terminal Myc tag (pCAGGS-Myc) or into the pET-28a vector with an N-terminal SUMO tag (pET-28a-SUMO). The DNA fragments encoding full-length human GSDMA (hGSDMA_FL) and murine GSDMA (mGSDMA_FL) were synthesized by Tsingke (China) and subsequently cloned into the pCAGGS-Myc vector. Eukaryotic expression plasmids for GSDMA_1–187 and GSDMA_1–247 were constructed through PCR amplification using the pGSDMA_FL plasmids as templates, followed by insertion into the pCAGGS-Myc vectors. The glutamine (Q) residues at positions 187 and 247 of GSDMA were mutated to alanine (A) through PCR amplification to generate GSDMA-Q187A, GSDMA-Q247A, and GSDMA-Q187A&Q247A. Eukaryotic expression plasmids encoding PEDV, HCoV-NL63, HCoV-HKU1, SARS-CoV-2, MERS-CoV, HCoV-OC43, MHV, PDCoV nsp5, and PDCoV nsp5_C144A were prepared as previously described [6,12,13,24]. The siRNAs targeting pGSDMA and mGSDMA, as well as the negative control siRNA (siNC) were synthesized by JTSBIO Co., Ltd (China). The siRNA sequences used were as follows (5’ to 3’): sipGSDMA (GCUUCUCCCUCCCAUUCUUTT and AAGAAUGGGAGGGAGAAGCTT); simGSDMA (GGAACUGAUCCUAACAGAATT and UUCUGUUAGGAUCAGUUCCTT). Transient transfection of expression plasmids and siRNAs was performed using Lipofectamine 3000 (Invitrogen, USA) according to the manufacturer’s instructions.

### Western blot analysis

Cells or tissues were lysed using a lysis buffer at pH 6.8, which contained 4% SDS, 3% DTT, 50 mM Tris-HCl, and 30% glycerin, supplemented with a protease inhibitor. Equal amounts of protein were loaded and separated by sodium dodecyl sulfate polyacrylamide gel electrophoresis (SDS-PAGE), followed by transfer onto polyvinylidene difluoride membranes. The membranes were blocked in Tris-buffered saline with 0.05% Tween 20 (TBST) containing 5% nonfat milk and subsequently incubated with the specified primary antibodies at 37°C for 3 h. After three washes with TBST, the membranes were incubated with horseradish peroxidase-conjugated secondary antibodies (Beyotime, China) for 1 h at room temperature. Following three additional washes with TBST, the bands on the membranes were visualized using enhanced chemiluminescence reagents (BIO-RAD, USA). The expression of β-Tubulin was utilized as a loading control.

### *In vitro* cleavage assay

PDCoV nsp5 and pGSDMA-SUMO proteins were purified using Ni-NTA columns, following established protocols [49–51]. Subsequently, the recombinant PDCoV nsp5 and pGSDMA-SUMO were combined in a buffer consisting of 20 mM HEPES, 50 mM NaCl, 0.4 mM EDTA, 30% glycerol, and 4 mM DTT at pH 8.0, resulting in a total volume of 100 μL. The reaction mixture was incubated at 37°C for 15 or 30 min, and the reactions were terminated by adding 1 × loading buffer prior to SDS-PAGE analysis.

### Sequence alignment

The ClustalW2 algorithm was utilized to perform amino acid sequence alignment for GSDMAs across various species. The results were visualized using ESPcript 3.2 (http://espript.ibcp.fr/ESPript/cgi-bin/ESPript.cgi).

### Cell cytotoxicity and viability

Cell death and viability were assessed using the CytoTox 96 Non-Radioactive Cytotoxicity Assay kit (Promega, USA) and the CellTiter-Glo Luminescent Cell Viability Assay kit (Promega) according to the manufacturer’s instructions.

### Enzyme-linked immunosorbent assay (ELISA)

The levels of porcine IL-1β, as well as murine IL-1β, TNF-α, and IL-6 in the supernatants were assessed using ELISA kits purchased from ABclonal. Additionally, the levels of porcine TNF-α, IL-6, and IL-8 in the supernatants were measured using ELISA kits obtained from Bioswamp (China). All procedures adhered to the manufacturer’s instructions.

### RNA extraction and quantitative real-time PCR (RT-qPCR)

Total RNA was extracted from cells or tissues using TRIzol reagent (Vazyme, China). Next, 1 μg of RNA was reverse-transcribed into cDNA using HiScript II Q RT SuperMix (Vazyme). The qPCR experiments were performed using SYBR Green PCR mix (Vazyme) and an ABI 7500 real-time PCR system (Applied Biosystems, USA). The specific primers are listed in S1 Table.

### Median tissue culture infectious dose (TCID_50_) assay

The TCID_50_ assays were performed as previously described [52]. In brief, cells were seeded into 96-well plates and subsequently infected with serial 10-fold dilutions of the viral sample, with eight replicates for each dilution. The plates were then incubated for 48–72 h before determining the virus titers. The titers of PDCoV, PEDV, and MHV were calculated using the Reed–Muench method and expressed as TCID_50_ per milliliter.

### Viral infections in mice

Specific-pathogen-free (SPF) C57BL/6J wild-type (WT) mice were procured from the Laboratory Animal Center of Huazhong Agricultural University. *Gsdma*^*-/-*^ mice were prepared as previously described [53]. All mice used in the experiments were naïve. No drug tests were performed. Mice were housed under a 12-hour light/dark cycle and provided ad libitum access to food and water. Four- to eight-week-old, sex-matched *Gsdma*^*-/-*^ mice and their WT littermates were intraperitoneally injected with MHV (1 × 10^6^ PFU/mouse) or DMEM as a mock infection. The survival rates and body weights of the mice were monitored daily following MHV infection. Sera from both mock-infected and MHV-infected mice were collected for ELISA analysis at 3 and 5 days post-infection (dpi), and liver tissues were harvested for RT-qPCR, histological, and ELISA analyses.

### Histological analysis

Liver tissues were fixed in 4% paraformaldehyde overnight and subsequently embedded in paraffin. Tissue damage was assessed through histological analysis using standard hematoxylin and eosin (H&E) staining. The stained tissue sections were examined under light microscopy, and representative images of these sections are presented in this study.

### Statistical analysis

GraphPad Prism 8 software was used for data analysis using a two-tailed unpaired *t*-test or one-way ANOVA. All statistical analyses were performed using GraphPad Software (GraphPad Inc., USA).

## Supporting Information

S1 Fig CoV nsp5 cleaves GSDMA across various species. (A) Recombinant SUMO-tagged pGSDMA (12.5 μg; pGSDMA-SUMO) protein was incubated with purified PDCoV nsp5 protein (1 μg) for 15 min. The protein levels of full-length pGSDMA-SUMO (pGSDMA_FL-SUMO) and cleaved pGSDMA-SUMO were analyzed using an anti-pGSDMA antibody. (B) HEK-293T cells were co-transfected with eukaryotic expression plasmids encoding HA-tagged PDCoV nsp5 (PDCoV nsp5-HA) and Myc-tagged pGSDMA_1–247 (pGSDMA_1–247-Myc). Cells were lysed at 48 hpt, and the expression levels of pGSDMA_1–247-Myc and cleaved pGSDMA_1–247-Myc were evaluated by immunoblotting using an anti-Myc antibody. β-Tubulin was used as a loading control. (C) Multiple sequence alignment of GSDMA from pig (p), human (h), monkey (mk), and bovine (b), as well as mouse (m) GSDMA1, was performed using the ClustalW2 algorithm and plotted using the ESPript program. Identical residues are highlighted with a red background, and similar residues are indicated in red. (D and E) Huh-7 cells (D) and iBMDMs (E) were transfected with eukaryotic expression plasmids encoding HA-tagged PDCoV nsp5 (PDCoV nsp5-HA). Cells were lysed at 48 h post-transfection (hpt), and the expression levels of endogenous full-length human GSDMA (hGSDMA_FL) and murine GSDMA (mGSDMA_FL), as well as cleaved hGSDMA and mGSDMA, were determined by immunoblotting using anti-GSDMA antibodies. (F to L) IPI-2I cells, THP-1 cells, and iBMDMs were transfected with eukaryotic expression plasmids encoding HA-tagged PEDV (F), HCoV-NL63 (G), HCoV-HKU1 (H), SARS-CoV-2 (I), MERS-CoV (J), HCoV-OC43 (K), or MHV nsp5 (L). Cells were lysed at 48 hpt, and the expression levels of endogenous GSDMA_FL and cleaved GSDMA were determined by immunoblotting using anti-GSDMA antibodies. β-Tubulin was used as a loading control.
(TIF)

S2 Fig GSDMA knockdown inhibits the pyroptosis induced by CoV infections. (A and B) IPI-2I cells (A) and iBMDMs (B) were transfected with GSDMA-specific siRNA (siGSDMA) or control siRNA (siNC). Cells were lysed at 36 hpt, and the expression of GSDMA_FL was determined by immunoblotting using anti-GSDMA antibodies. β-Tubulin was used as a loading control. (C to E) IPI-2I cells (C and D) and iBMDMs (E) were transfected with siGSDMA or siNC, followed by infection with PDCoV (C), PEDV (D), and MHV (E). At the indicated time points, LDH release, ATP cell viability, and IL-1β protein levels were analyzed using assay kits. Data analysis was performed using one-way ANOVA. Values are shown as the mean ± SD from three independent experiments. **, p < 0.01; ***, p < 0.001.
(TIF)

S3 Fig GSDMA knockdown inhibits the inflammatory responses induced by CoV infections. IPI-2I cells (A and B) and iBMDMs (C) were transfected with siGSDMA or siNC, followed by infection with PDCoV (A), PEDV (B), and MHV (C). Cell supernatants were collected, and the protein levels of TNF-α, IL-6, and/or IL-8 were assessed using ELISA assays. Data analysis was performed using one-way ANOVA. Values are shown as the mean ± SD from three independent experiments. **, *p* < 0.01; ***, *p* < 0.001.
(TIF)

S4 Fig GSDMA_1–247 enhances the activation of IFN-β signaling more significantly than GSDMA_FL. (A) IPI-2I cells were transfected with eukaryotic expression plasmids encoding pGSDMA_1–247, pGSDMA_1–187, and pGSDMA_FL. The relative mRNA levels of *IFNB1* and ISGs (*ISG15*, *IFIT1*, and *IFIT2*) were measured by RT-qPCR. *ACTB* was used as the internal control. (B) iBMDMs were transfected with eukaryotic expression plasmids encoding mGSDMA_1–247, mGSDMA_1–187, and mGSDMA_FL. The relative mRNA levels of *Ifnb1* and ISGs (*Isg15*, *Ifit1*, and *Ifit2*) were measured by RT-qPCR. *Gapdh* was used as the internal control. Data analysis was performed using one-way ANOVA. Values are shown as the mean ± SD from three independent experiments. **, *p* < 0.01; ***, *p* < 0.001.
(TIF)

S5 Fig *Gsdma*^*-/-*^ mice show milder inflammatory symptoms under MHV infection at 3dpi. (A) Liver tissues from WT or *Gsdma*^*-/-*^ mice, either mock-infected or infected with MHV, were harvested and lysed for immunoblotting. Endogenous GSDMA_FL and cleaved GSDMA were assessed using an anti-GSDMA antibody. β-Tubulin was used as a loading control. (B to D) Liver tissues from mice euthanized at 3 dpi were analyzed for viral titers using L929 cells, as well as for viral RNA copy number (B), gross lesions (C), and pathological changes (D). (E to G) Sera and liver tissues from mice euthanized at 3 dpi and/or 5 dpi were collected to assess the protein levels of IL-1β (E), TNF-α (F), and IL-6 (G) using ELISA assays. (B and E to G) Data analysis was performed using Student’s *t*-test (B) or one-way ANOVA (E to G). Values are shown as the mean ± SD. *, *p* < 0.05; **, *p* < 0.01; ***, *p* < 0.001.
(TIF)

S6 Fig PDCoV nsp5 promotes cell death and inflammatory responses. IPI-2I cells were transfected with eukaryotic expression plasmids encoding PDCoV nsp5. (A) At 48 hpt, LDH release and ATP-based cell viability were measured. (B) The relative mRNA levels of *TNF*, *IL6*, and *CXCL8* were analyzed by RT-qPCR. *ACTB* was used as the internal control. Data analysis was performed using Student’s t-test. Values are shown as the mean ± SD from three independent experiments. ***, *p* < 0.001.
(TIF)

S7 Fig Validation of a monoclonal antibody against pGSDMA. (A) SDS-PAGE followed by Coomassie Brilliant Blue staining of purified recombinant pGSDMA protein used for immunization. (B) Western blot analysis of pGSDMA in HEK-293T cells transfected with the eukaryotic expression plasmid pCAGGS-pGSDMA-Myc. Blots were probed with an anti-Myc monoclonal antibody or the anti-pGSDMA mouse monoclonal antibody. (C) Western blot analysis of endogenous pGSDMA in IPI-2I cells using the anti-pGSDMA mouse monoclonal antibody.
(TIF)

S1 Table Primers used for RT-qPCR. (XLSX)

S1 Data Excel spreadsheet containing the underlying numerical data for Figures 1C, 3B-3H, 4A-4F, 5B, 5C, 5E, 5H, 5I, S2C-S2E, S3A-S3C, S4A, S4B, S5B, S5E-S5G, and S6A-S6B, provided in separate worksheets. (XLSX)

S1 Raw Images Uncropped raw images for western blot and SDS-PAGE analyses. (ZIP)
