# Supplementary material for: Coronavirus Nsp5‑mediated dual‑site cleavage of GSDMA modifies its antiviral and proinflammatory functions
Source: PLoS Pathog. 2026 Sep 16;22(9):e1014602. doi: 10.1371/journal.ppat.1014602 (PMC13600616; doi:10.1371/journal.ppat.1014602)
Supplement: Supplementary file 1 [file ppat.1014602.s010.zip › S1 Raw Images/Raw images legends.docx]

**Raw images legends**

**Fig 1A**

pGSDMA_FL and Cleaved pGSDMA: Bio‑Rad ECL was used for chemiluminescent detection, with exposure performed on a ChemiDoc MP Imaging System, and the protein marker was from Epizyme Biomedical (left) or Abclonal (right).

HA (PDCoV nsp5): Bio‑Rad ECL was used for chemiluminescent detection, with exposure performed on a ChemiDoc MP Imaging System, and the protein marker was from Epizyme Biomedical (left) or Abclonal (right).

β-Tubulin: Bio‑Rad ECL was used for chemiluminescent detection, with exposure performed on a ChemiDoc MP Imaging System, and the protein marker was from Epizyme Biomedical (left) or Abclonal (right).

**Fig 1B**

pGSDMA_FL-SUMO and Cleaved pGSDMA-SUMO: Bio‑Rad ECL was used for chemiluminescent detection, with exposure performed on a ChemiDoc MP Imaging System, and the protein marker was from Epizyme Biomedical (left) or Abclonal (right).

**Fig 1D**

pGSDMA_FL-Myc and Cleaved pGSDMA-Myc: Bio‑Rad ECL was used for chemiluminescent detection, with exposure performed on a ChemiDoc MP Imaging System, and the protein marker was from Epizyme Biomedical.

HA (PDCoV nsp5): Bio‑Rad ECL was used for chemiluminescent detection, with exposure performed on a ChemiDoc MP Imaging System, and the protein marker was from Epizyme Biomedical.

β-Tubulin: Bio‑Rad ECL was used for chemiluminescent detection, with exposure performed on a ChemiDoc MP Imaging System, and the protein marker was from Epizyme Biomedical.

**Fig 1E**

pGSDMA_FL-Myc and Cleaved pGSDMA-Myc: Bio‑Rad ECL was used for chemiluminescent detection, with exposure performed on a ChemiDoc MP Imaging System, and the protein marker was from Epizyme Biomedical.

HA (PEDV nsp5): Bio‑Rad ECL was used for chemiluminescent detection, with exposure performed on a ChemiDoc MP Imaging System, and the protein marker was from Epizyme Biomedical.

β-Tubulin: Bio‑Rad ECL was used for chemiluminescent detection, with exposure performed on a ChemiDoc MP Imaging System, and the protein marker was from Epizyme Biomedical.

**Fig 1F**

mGSDMA_FL-Myc and Cleaved mGSDMA-Myc: Bio‑Rad ECL was used for chemiluminescent detection, with exposure performed on a ChemiDoc MP Imaging System, and the protein marker was from Epizyme Biomedical.

HA (MHV nsp5): Bio‑Rad ECL was used for chemiluminescent detection, with exposure performed on a ChemiDoc MP Imaging System, and the protein marker was from Epizyme Biomedical.

β-Tubulin: Bio‑Rad ECL was used for chemiluminescent detection, with exposure performed on a ChemiDoc MP Imaging System, and the protein marker was from Epizyme Biomedical.

**Fig 2A**

pGSDMA_FL and Cleaved pGSDMA: Bio‑Rad ECL was used for chemiluminescent detection, with exposure performed on a ChemiDoc MP Imaging System, and the protein marker was from Epizyme Biomedical.

PDCoV N: Bio‑Rad ECL was used for chemiluminescent detection, with exposure performed on a ChemiDoc MP Imaging System, and the protein marker was from Epizyme Biomedical.

β-Tubulin: Bio‑Rad ECL was used for chemiluminescent detection, with exposure performed on a ChemiDoc MP Imaging System, and the protein marker was from Epizyme Biomedical.

**Fig 2B**

pGSDMA_FL and Cleaved pGSDMA: Bio‑Rad ECL was used for chemiluminescent detection, with exposure performed on a ChemiDoc MP Imaging System, and the protein marker was from Epizyme Biomedical.

PDCoV N: Bio‑Rad ECL was used for chemiluminescent detection, with exposure performed on a ChemiDoc MP Imaging System, and the protein marker was from Epizyme Biomedical.

β-Tubulin: Bio‑Rad ECL was used for chemiluminescent detection, with exposure performed on a ChemiDoc MP Imaging System, and the protein marker was from Epizyme Biomedical.

**Fig 2C**

pGSDMA_FL and Cleaved pGSDMA: Bio‑Rad ECL was used for chemiluminescent detection, with exposure performed on a ChemiDoc MP Imaging System, and the protein marker was from Epizyme Biomedical.

PEDV N: Bio‑Rad ECL was used for chemiluminescent detection, with exposure performed on a ChemiDoc MP Imaging System, and the protein marker was from Epizyme Biomedical.

β-Tubulin: Bio‑Rad ECL was used for chemiluminescent detection, with exposure performed on a ChemiDoc MP Imaging System, and the protein marker was from Epizyme Biomedical.

**Fig 2D**

pGSDMA_FL and Cleaved pGSDMA: Bio‑Rad ECL was used for chemiluminescent detection, with exposure performed on a ChemiDoc MP Imaging System, and the protein marker was from Epizyme Biomedical.

PEDV N: Bio‑Rad ECL was used for chemiluminescent detection, with exposure performed on a ChemiDoc MP Imaging System, and the protein marker was from Epizyme Biomedical.

β-Tubulin: Bio‑Rad ECL was used for chemiluminescent detection, with exposure performed on a ChemiDoc MP Imaging System, and the protein marker was from Epizyme Biomedical.

**Fig 2E**

mGSDMA_FL and Cleaved mGSDMA: Bio‑Rad ECL was used for chemiluminescent detection, with exposure performed on a ChemiDoc MP Imaging System, and the protein marker was from Epizyme Biomedical.

MHV N: Bio‑Rad ECL was used for chemiluminescent detection, with exposure performed on a ChemiDoc MP Imaging System, and the protein marker was from Epizyme Biomedical.

β-Tubulin: Bio‑Rad ECL was used for chemiluminescent detection, with exposure performed on a ChemiDoc MP Imaging System, and the protein marker was from Epizyme Biomedical.

**Fig 2F**

mGSDMA_FL and Cleaved mGSDMA: Bio‑Rad ECL was used for chemiluminescent detection, with exposure performed on a ChemiDoc MP Imaging System, and the protein marker was from Epizyme Biomedical.

MHV N: Bio‑Rad ECL was used for chemiluminescent detection, with exposure performed on a ChemiDoc MP Imaging System, and the protein marker was from Epizyme Biomedical.

β-Tubulin: Bio‑Rad ECL was used for chemiluminescent detection, with exposure performed on a ChemiDoc MP Imaging System, and the protein marker was from Epizyme Biomedical.

**S1A Fig**

pGSDMA_FL-SUMO and Cleaved pGSDMA-SUMO: Bio‑Rad ECL was used for chemiluminescent detection, with exposure performed on a ChemiDoc MP Imaging System, and the protein marker was from Epizyme Biomedical.

**S1B Fig**

pGSDMA_1-247-Myc and Cleaved pGSDMA-Myc: Bio‑Rad ECL was used for chemiluminescent detection, with exposure performed on a ChemiDoc MP Imaging System, and the protein marker was from Epizyme Biomedical.

HA (PDCoV nsp5): Bio‑Rad ECL was used for chemiluminescent detection, with exposure performed on a ChemiDoc MP Imaging System, and the protein marker was from Epizyme Biomedical.

β-Tubulin: Bio‑Rad ECL was used for chemiluminescent detection, with exposure performed on a ChemiDoc MP Imaging System, and the protein marker was from Epizyme Biomedical.

**S1D Fig**

hGSDMA_FL and Cleaved hGSDMA: Bio‑Rad ECL was used for chemiluminescent detection, with exposure performed on a ChemiDoc MP Imaging System, and the protein marker was from Epizyme Biomedical.

HA (PDCoV nsp5): Bio‑Rad ECL was used for chemiluminescent detection, with exposure performed on a ChemiDoc MP Imaging System, and the protein marker was from Epizyme Biomedical.

β-Tubulin: Bio‑Rad ECL was used for chemiluminescent detection, with exposure performed on a ChemiDoc MP Imaging System, and the protein marker was from Epizyme Biomedical.

**S1E Fig**

mGSDMA_FL and Cleaved mGSDMA: Bio‑Rad ECL was used for chemiluminescent detection, with exposure performed on a ChemiDoc MP Imaging System, and the protein marker was from Epizyme Biomedical.

HA (PDCoV nsp5): Bio‑Rad ECL was used for chemiluminescent detection, with exposure performed on a ChemiDoc MP Imaging System, and the protein marker was from Epizyme Biomedical.

β-Tubulin: Bio‑Rad ECL was used for chemiluminescent detection, with exposure performed on a ChemiDoc MP Imaging System, and the protein marker was from Epizyme Biomedical.

**S1F Fig**

pGSDMA_FL and Cleaved pGSDMA: Bio‑Rad ECL was used for chemiluminescent detection, with exposure performed on a ChemiDoc MP Imaging System, and the protein marker was from Epizyme Biomedical.

HA (PEDV nsp5): Bio‑Rad ECL was used for chemiluminescent detection, with exposure performed on a ChemiDoc MP Imaging System, and the protein marker was from Epizyme Biomedical.

β-Tubulin: Bio‑Rad ECL was used for chemiluminescent detection, with exposure performed on a ChemiDoc MP Imaging System, and the protein marker was from Epizyme Biomedical.

**S1G Fig**

hGSDMA_FL and Cleaved hGSDMA: Bio‑Rad ECL was used for chemiluminescent detection, with exposure performed on a ChemiDoc MP Imaging System, and the protein marker was from Epizyme Biomedical.

HA (HCoV-NL63 nsp5): Bio‑Rad ECL was used for chemiluminescent detection, with exposure performed on a ChemiDoc MP Imaging System, and the protein marker was from Epizyme Biomedical.

β-Tubulin: Bio‑Rad ECL was used for chemiluminescent detection, with exposure performed on a ChemiDoc MP Imaging System, and the protein marker was from Epizyme Biomedical.

**S1H Fig**

hGSDMA_FL and Cleaved hGSDMA: Bio‑Rad ECL was used for chemiluminescent detection, with exposure performed on a ChemiDoc MP Imaging System, and the protein marker was from Epizyme Biomedical.

HA (HCoV-HKU1 nsp5): Bio‑Rad ECL was used for chemiluminescent detection, with exposure performed on a ChemiDoc MP Imaging System, and the protein marker was from Epizyme Biomedical.

β-Tubulin: Bio‑Rad ECL was used for chemiluminescent detection, with exposure performed on a ChemiDoc MP Imaging System, and the protein marker was from Epizyme Biomedical.

**S1I Fig**

hGSDMA_FL and Cleaved hGSDMA: Bio‑Rad ECL was used for chemiluminescent detection, with exposure performed on a ChemiDoc MP Imaging System, and the protein marker was from Epizyme Biomedical.

HA (SARS-CoV-2 nsp5): Bio‑Rad ECL was used for chemiluminescent detection, with exposure performed on a ChemiDoc MP Imaging System, and the protein marker was from Epizyme Biomedical.

β-Tubulin: Bio‑Rad ECL was used for chemiluminescent detection, with exposure performed on a ChemiDoc MP Imaging System, and the protein marker was from Epizyme Biomedical.

**S1J Fig**

hGSDMA_FL and Cleaved hGSDMA: Bio‑Rad ECL was used for chemiluminescent detection, with exposure performed on a ChemiDoc MP Imaging System, and the protein marker was from Epizyme Biomedical.

HA (MERS-CoV nsp5): Bio‑Rad ECL was used for chemiluminescent detection, with exposure performed on a ChemiDoc MP Imaging System, and the protein marker was from Epizyme Biomedical.

β-Tubulin: Bio‑Rad ECL was used for chemiluminescent detection, with exposure performed on a ChemiDoc MP Imaging System, and the protein marker was from Epizyme Biomedical.

**S1K Fig**

hGSDMA_FL and Cleaved hGSDMA: Bio‑Rad ECL was used for chemiluminescent detection, with exposure performed on a ChemiDoc MP Imaging System, and the protein marker was from Epizyme Biomedical.

HA (HCoV-OC43 nsp5): Bio‑Rad ECL was used for chemiluminescent detection, with exposure performed on a ChemiDoc MP Imaging System, and the protein marker was from Epizyme Biomedical.

β-Tubulin: Bio‑Rad ECL was used for chemiluminescent detection, with exposure performed on a ChemiDoc MP Imaging System, and the protein marker was from Epizyme Biomedical.

**S1L Fig**

mGSDMA_FL and Cleaved mGSDMA: Bio‑Rad ECL was used for chemiluminescent detection, with exposure performed on a ChemiDoc MP Imaging System, and the protein marker was from Epizyme Biomedical.

HA (MHV nsp5): Bio‑Rad ECL was used for chemiluminescent detection, with exposure performed on a ChemiDoc MP Imaging System, and the protein marker was from Epizyme Biomedical.

β-Tubulin: Bio‑Rad ECL was used for chemiluminescent detection, with exposure performed on a ChemiDoc MP Imaging System, and the protein marker was from Epizyme Biomedical.

**S2A Fig**

pGSDMA: Bio‑Rad ECL was used for chemiluminescent detection, with exposure performed on a ChemiDoc MP Imaging System, and the protein marker was from Abclonal.

β-Tubulin: Bio‑Rad ECL was used for chemiluminescent detection, with exposure performed on a ChemiDoc MP Imaging System, and the protein marker was from Abclonal.

**S2B Fig**

mGSDMA: Bio‑Rad ECL was used for chemiluminescent detection, with exposure performed on a ChemiDoc MP Imaging System, and the protein marker was from Abclonal.

β-Tubulin: Bio‑Rad ECL was used for chemiluminescent detection, with exposure performed on a ChemiDoc MP Imaging System, and the protein marker was from Abclonal.

**S5A Fig**

GSDMA_FL and Cleaved GSDMA: Bio‑Rad ECL was used for chemiluminescent detection, with exposure performed on a ChemiDoc MP Imaging System, and the protein marker was from Epizyme Biomedical.

MHV N: Bio‑Rad ECL was used for chemiluminescent detection, with exposure performed on a ChemiDoc MP Imaging System, and the protein marker was from Epizyme Biomedical.

β-Tubulin: Bio‑Rad ECL was used for chemiluminescent detection, with exposure performed on a ChemiDoc MP Imaging System, and the protein marker was from Epizyme Biomedical.

**S7A Fig**

pGSDMA-SUMO: Coomassie staining was used for protein visualization, with exposure performed on a ChemiDoc MP Imaging System, and the protein marker was from Epizyme Biomedical.

**S7B Fig**

pGSDMA: Bio‑Rad ECL was used for chemiluminescent detection, with exposure performed on a ChemiDoc MP Imaging System, and the protein marker was from Epizyme Biomedical.

**S7C Fig**

pGSDMA: Bio‑Rad ECL was used for chemiluminescent detection, with exposure performed on a ChemiDoc MP Imaging System, and the protein marker was from Epizyme Biomedical.
