# Supplementary material for: Coronavirus Nsp5‑mediated dual‑site cleavage of GSDMA modifies its antiviral and proinflammatory functions
Source: PLoS Pathog. 2026 Sep 16;22(9):e1014602. doi: 10.1371/journal.ppat.1014602 (PMC13600616; doi:10.1371/journal.ppat.1014602)

Fig. 1A

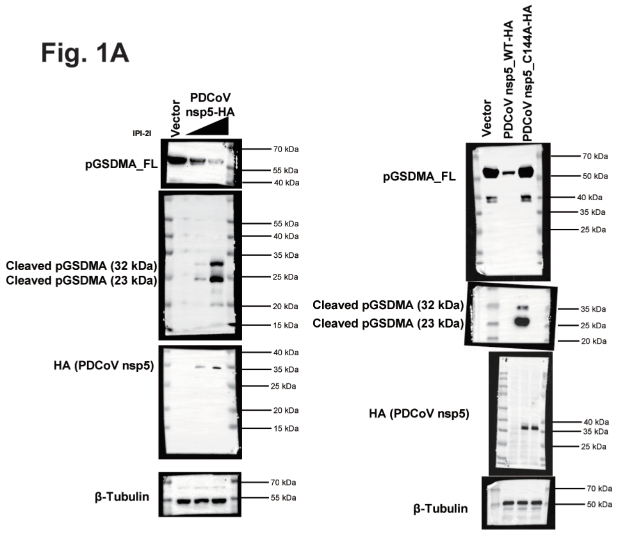

Fig. 1B

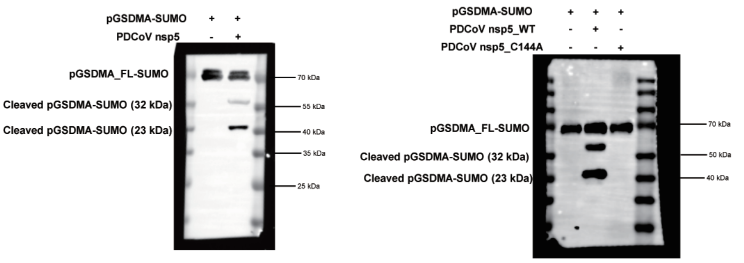

Fig. 1D

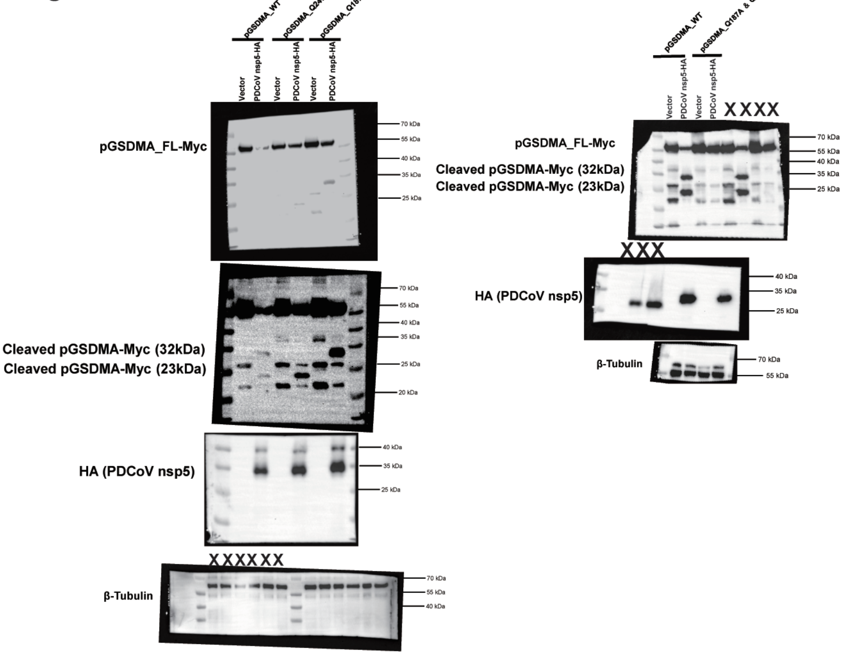

Fig. 1E

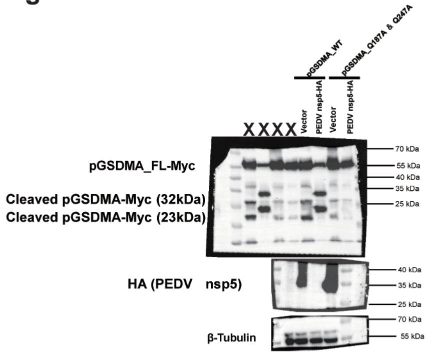

Fig. 1F

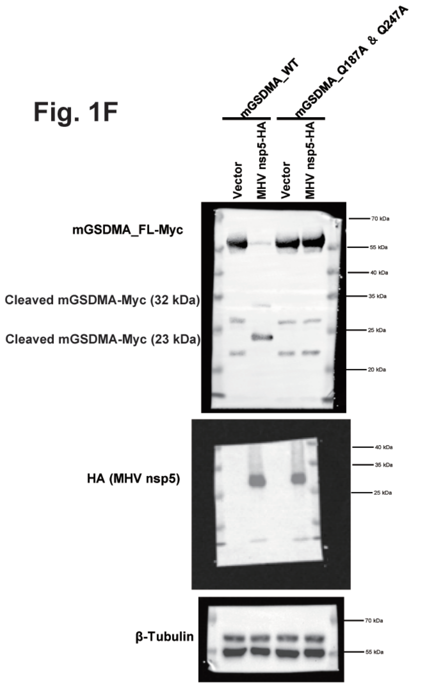

Fig. 2A

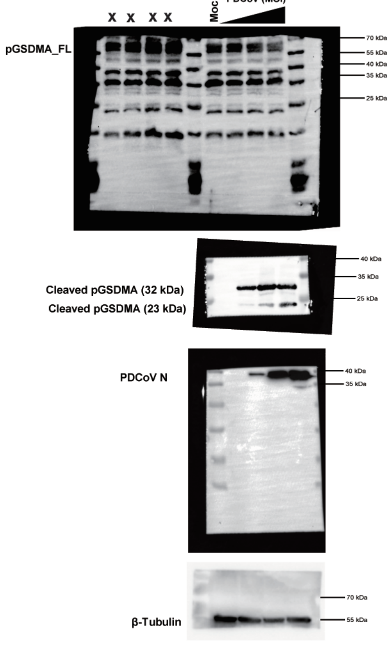

Fig. 2B

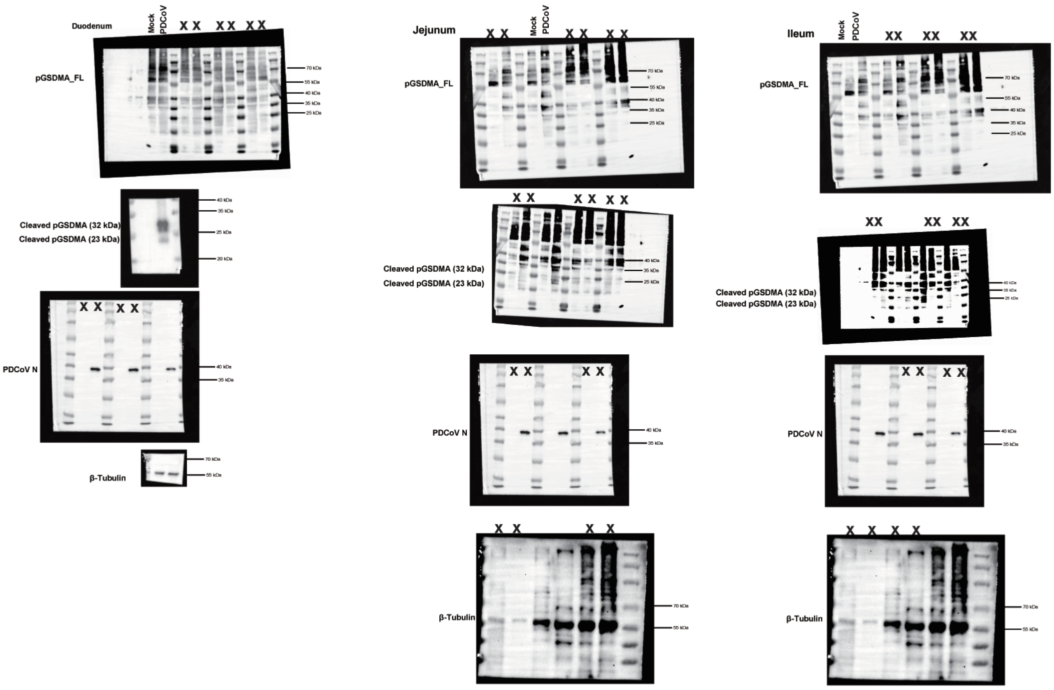

Fig. 2C

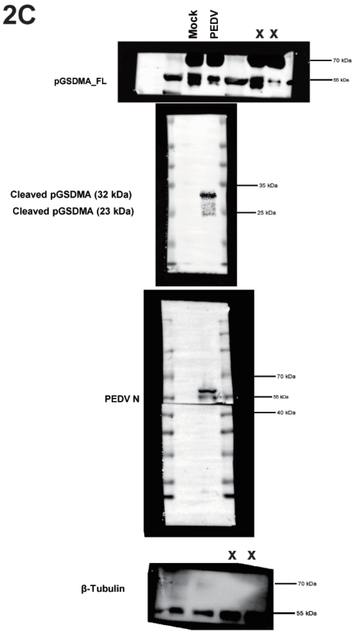

Fig. 2D

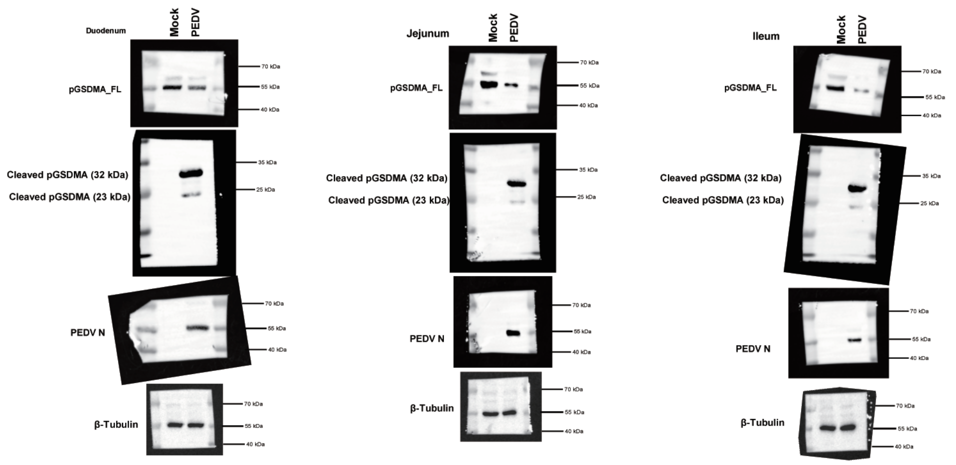

Fig. 2E

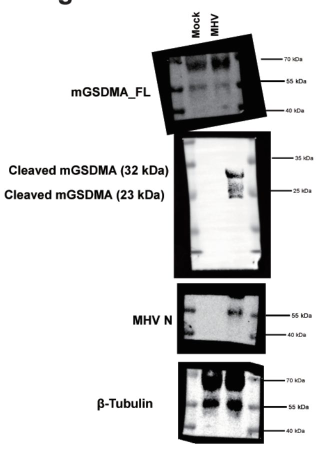

Fig. 2F

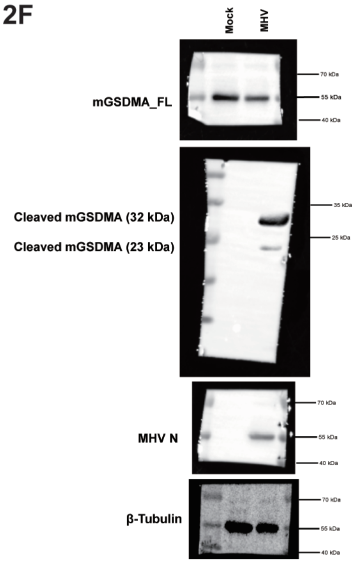

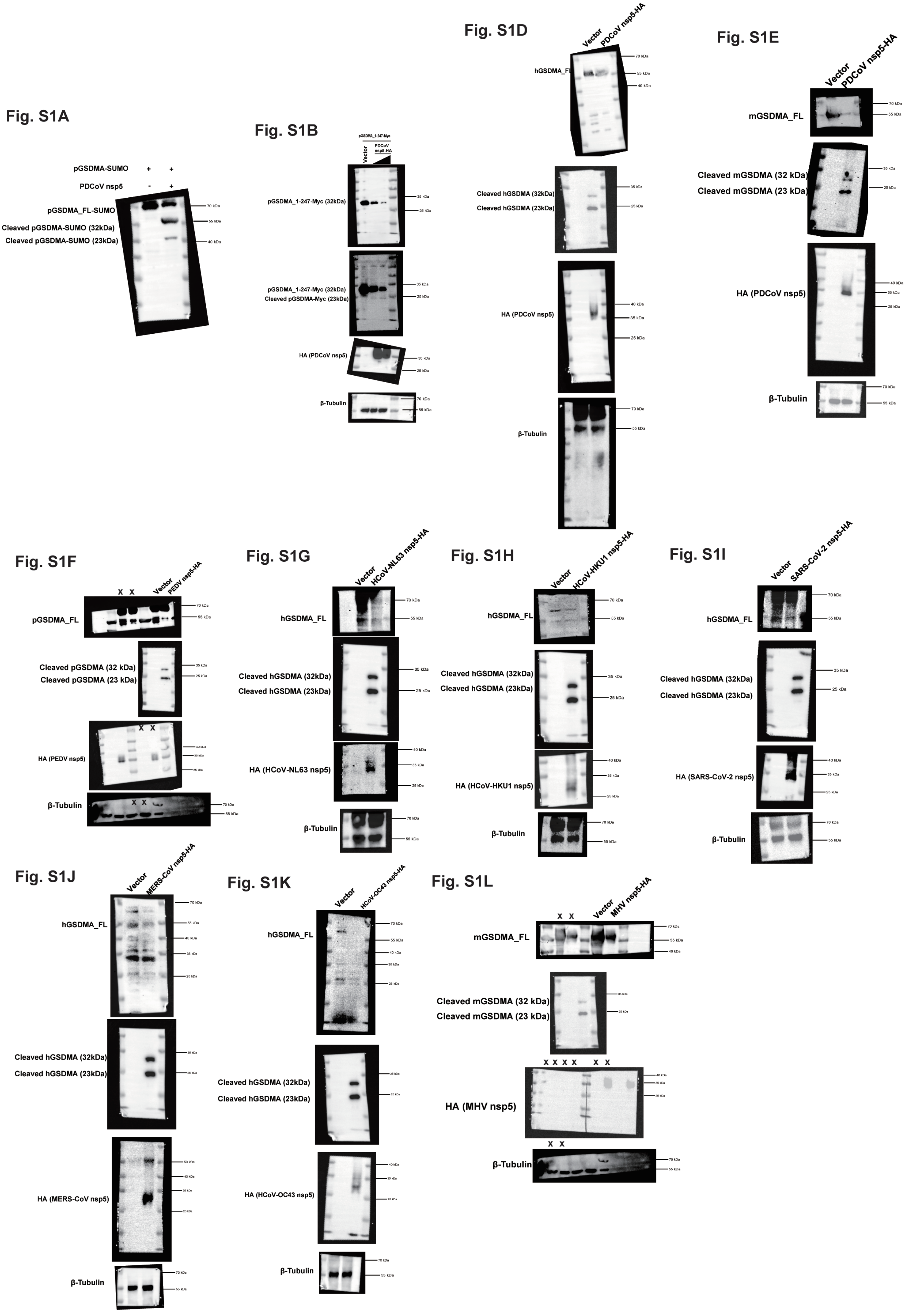
Fig. S1A
pGSDMA-SUMO
PDCoV nsp5
pGSDMA_FL-SUMO
Cleaved pGSDMA-SUMO (32kDa)
Cleaved pGSDMA-SUMO (23kDa)
Fig. S1B
pGSDMA_1-247-Myc
Vector
PDCoV nsp5-HA
pGSDMA_1-247-Myc (32kDa)
Cleaved pGSDMA-Myc (23kDa)
HA (PDCoV nsp5)
β-Tubulin
Fig. S1D
Vector
PDCoV nsp5-HA
hGSDMA_FL
Cleaved hGSDMA (32kDa)
Cleaved hGSDMA (23kDa)
HA (PDCoV nsp5)
β-Tubulin
Fig. S1E
Vector
PDCoV nsp5-HA
mGSDMA_FL
Cleaved mGSDMA (32 kDa)
Cleaved mGSDMA (23 kDa)
HA (PDCoV nsp5)
β-Tubulin
Fig. S1F
Vector
PEDV nsp5-HA
pGSDMA_FL
Cleaved pGSDMA (32 kDa)
Cleaved pGSDMA (23 kDa)
HA (PEDV nsp5)
β-Tubulin
Fig. S1G
Vector
HCoV-NL63 nsp5-HA
hGSDMA_FL
Cleaved hGSDMA (32kDa)
Cleaved hGSDMA (23kDa)
HA (HCoV-NL63 nsp5)
β-Tubulin
Fig. S1H
Vector
HCoV-HKU1 nsp5-HA
hGSDMA_FL
Cleaved hGSDMA (32kDa)
Cleaved hGSDMA (23kDa)
HA (HCoV-HKU1 nsp5)
β-Tubulin
Fig. S1I
Vector
SARS-CoV-2 nsp5-HA
hGSDMA_FL
Cleaved hGSDMA (32kDa)
Cleaved hGSDMA (23kDa)
HA (SARS-CoV-2 nsp5)
β-Tubulin
Fig. S1J
Vector
MERS-CoV nsp5-HA
hGSDMA_FL
Cleaved hGSDMA (32kDa)
Cleaved hGSDMA (23kDa)
HA (MERS-CoV nsp5)
β-Tubulin
Fig. S1K
Vector
HCoV-OC43 nsp5-HA
hGSDMA_FL
Cleaved hGSDMA (32kDa)
Cleaved hGSDMA (23kDa)
HA (HCoV-OC43 nsp5)
β-Tubulin
Fig. S1L
Vector
MHV nsp5-HA
mGSDMA_FL
Cleaved mGSDMA (32 kDa)
Cleaved mGSDMA (23 kDa)
HA (MHV nsp5)
β-Tubulin

Fig. S2A

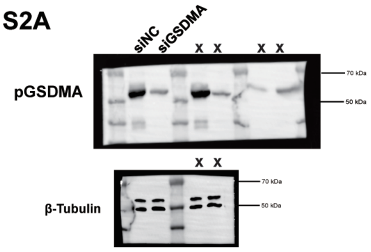

Fig. S2B

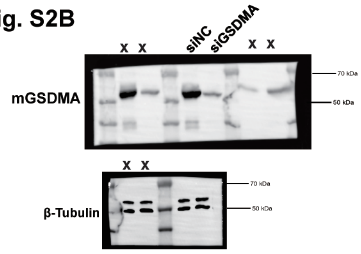

Fig. S5A

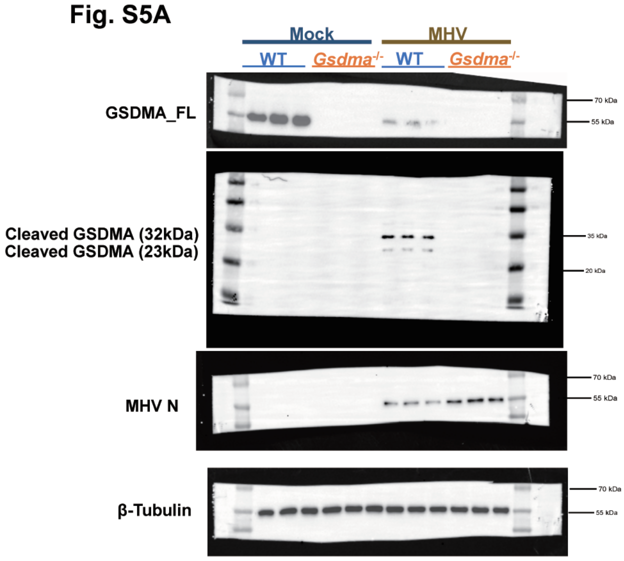

Fig. S7A

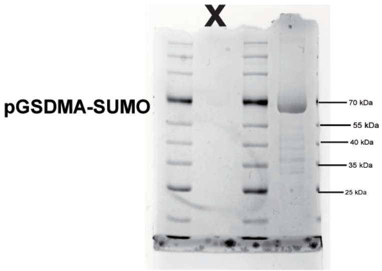

Fig. S7B

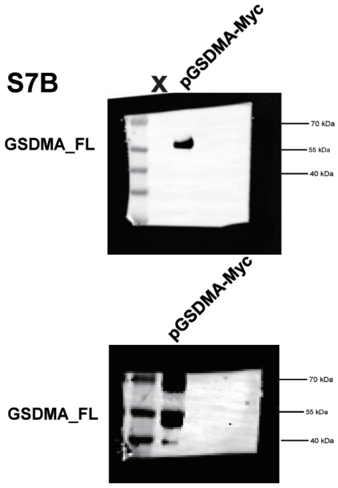

Fig. S7C

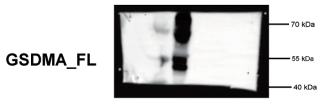

Supplement: Supplementary file 1 [file ppat.1014602.s010.zip › S1 Raw Images/S1_raw_images.pdf]
